# A Long-Acting Farnesoid X Receptor (FXR) Agonist Prevents Fibrosis

**DOI:** 10.3390/biom16101443

**Published:** 2026-10-03

**Authors:** Ashrafi Sultana, Grace Bybee, Anup S. Pathania, Weimin Wang, Nam Thai Hoang Le, Brandon W. Hanson, Christopher D. Bauer, Deanna D. Mosley, Howard E. Gendelman, Samuel M. Cohen, Todd A. Wyatt, Larisa Y. Poluektova, Benson J. Edagwa, Natalia A. Osna

**Affiliations:** 1 Medical Sciences Interdepartmental Area Program, University of Nebraska Medical Center, Omaha, NE 68198, USA; 2 Department of Pharmacology and Experimental Neuroscience, University of Nebraska Medical Center, Omaha, NE 68198, USAhegendel@unmc.edu (H.E.G.); lpoluekt@unmc.edu (L.Y.P.); 3 Department of Environmental, Agricultural and Occupational Health, University of Nebraska Medical Center, Omaha, NE 68198, USAtwyatt@unmc.edu (T.A.W.); 4 Department of Pathology, Microbiology and Immunology, and Buffett Cancer Center, University of Nebraska Medical Center, Omaha, NE 68198, USA; scohen@unmc.edu; 5 Department of Internal Medicine, Pulmonary, Critical Care and Sleep Medicine Division, University of Nebraska Medical Center, Omaha, NE 68198, USA; 6 Veterans Affairs Nebraska-Western Iowa Health Care System, Research Service, 4101 Woolworth Avenue, Omaha, NE 68105, USA

**Keywords:** vonafexor, farnesoid X receptor agonist, long-acting prodrug, lung and liver fibrosis, hepatitis B

## Abstract

Despite effective vaccines and antiviral therapies, patients with chronic hepatitis B (CHB) remain at risk of developing progressive liver fibrosis and cirrhosis. Vonafexor (VON), a second-generation synthetic non-steroidal, non-bile acid farnesoid X receptor agonist, has been shown to prevent liver fibrosis and target the HBV cccDNA reservoir. However, adherence to daily oral therapy is an established barrier to successful treatment of chronic infections. To overcome this barrier, VON was transformed into a long-acting (LA) prodrug nanosuspension. The LA prodrug formulation suppressed αSMA, TGFβ, and Col1A1 gene expression in the LX2 hepatic stellate cell line. The formulation was well tolerated and prevented liver and lung damage in a carbon tetrachloride-induced chronic liver injury model. The promising preclinical data support further development of LA VON prodrugs as potential treatments for liver and lung fibrosis.

## 1. Introduction

Chronic hepatitis B (CHB) induced by hepatotropic viruses often progresses to end-stage liver disease. Liver fibrosis is the initial reversible stage of disease that precedes the development of liver cirrhosis and hepatocellular carcinoma (HCC). About 20 to 30% of inactive Hepatis B virus (HBV carriers with normal alanine transaminase (ALT) levels and low viral loads (10^4^ copies of HBV DNA). These patients may have underlying fibrosis, as identified by liver biopsy. Normal ALT is an imperfect marker of inflammation-associated liver health, and fibrosis can persist or develop even with low levels of viral replication [1]. In addition to the risk of cirrhosis, CHB infection initiates metabolic dysfunction-associated steatohepatitis (MASH), which is characterized by chronic, progressive liver inflammation, excess fat accumulation (steatosis), and hepatocyte ballooning. This can often lead to fibrosis and cirrhosis. The condition is driven by insulin resistance, obesity, type 2 diabetes, high blood lipid levels, hepatocyte injury, and inflammation. Disease can progress in patients even with demonstrated low viral loads. However, while persistent viremia can affect progression to fibrosis, higher viral loads and excessive alcohol consumption can accelerate advanced liver fibrosis [2]. The annual incidence of cirrhosis, as reported by ClinicalTrials.gov, is 2–10% in patients with CHB. There is also a 3–6% annual incidence of HCC. In addition to antiviral therapy, affected patients require long-term antifibrotic treatment. Such treatments target hepatic stellate cells (HSC), which promote dysfunctional extracellular matrix. 

The Farnesoid X Receptor (FXR; also known as NR1H4) affects liver cirrhosis by acting as a protective regulator that inhibits fibrosis, reduces bile acid toxicity, and suppresses liver inflammation. Activating FXR with agonists that include obeticholic acid (OCA) and fexaramine is known to regulate bile acid, lipid, and glucose metabolism. Each can attenuate liver fibrosis, making them targets for controlling chronic liver diseases that progress to cirrhosis. FXR is highly expressed in the liver and intestine and serves to regulate cholesterol and bile acid homeostasis, lipogenesis, hepatic gluconeogenesis, inflammation, and fibrosis [3]. FXR agonists reduce liver fibrosis. This is mediated through direct and indirect effects on HSC, the primary effectors of liver fibrosis and cirrhosis [4]. The activated HSC proliferate and secrete excessive extracellular matrix (ECM) components, such as collagen, leading to fibrosis, scar tissue formation, and ultimately cirrhosis. As previously demonstrated, the natural bile-derived FXR agonist OCA prevents HSC activation. This occurs by stabilizing perilipin 1 in lipid droplets [5]. OCA suppresses oxidative stress, downregulates apoptosis and inflammasome activation, and slows fibrosis during MASH treatment [3,6]. OCA is an FDA-approved drug for treating primary biliary cholangitis. However, severe pruritus can occur as an adverse event in some patients receiving OCA. Such events can impact treatment adherence and therapeutic efficacy [7]. This facilitated the development of non-steroidal FXR agonists, including vonafexor (VON), which have improved liver injury in mouse models of the disease. VON treatment has also significantly improved MASH parameters in phase 1 and 2 clinical trials [3]. The promising clinical findings underscore the potential of daily oral VON in preventing liver fibrosis [7,8,9].

Metabolic alterations promote fibrosis in chronic HBV infection. In addition to its promising anti-HBV activity [10], VON’s ability to target key pathways in HSC activation offers a promising strategy for combating liver fibrosis. However, to realize its full potential, lifelong treatment is required to improve liver health in patients with chronic HBV infection. Adherence to therapy is well established as the key to successful long-term treatment of chronic infections. Simplification of therapy has been associated with significant improvements in patient satisfaction and treatment outcomes. This proof-of-concept study aimed to evaluate the potential of a non-oral, long-acting (LA) VON prodrug formulation to treat fibrosis in preclinical disease models. 

In this study, VON was transformed into an LA-injectable prodrug nanosuspension (NM2VON). The formulation was stable for over a year. The formulation attenuated liver and lung fibrosis in a mouse model of carbon tetrachloride (CCl_4_)-induced liver damage. Three monthly intramuscular injections of NM2VON at 200 mg/kg were well tolerated. HSC demonstrated that NM2VON significantly suppressed alpha smooth muscle actin (αSMA), transforming growth factor beta (TGFβ), and collagen type I alpha 1 chain (Col1A1) gene expression when compared against the parent drug control. Intramuscular injections of NM2VON in Sprague Dawley (SD) rats were well tolerated, with no identified toxicities. These promising proof-of-concept findings illustrate the potential of the LA NM2VON formulation for the prevention and treatment of liver and lung fibrosis.

## 2. Materials and Methods

### 2.1. Reagents

VON was purchased from Ambeed Inc. (Buffalo Grove, IL, USA). 1-octadecanol, triethylamine (Et_3_N), Hexafluorophosphate Azabenzotriazole Tetramethyl Uronium (HATU), 2,2′-Dimethylformamide (DMF), Chloroform (CHCl_3_), Tween 20, polyethylene glycol 3350 (PEG 3350), 2,5-diphenyltetrazolium bromide (MTT), dimethyl sulfoxide (DMSO), potassium chloride, sodium chloride, sodium sulphate, sodium carbonate, and ciprofloxacin were purchased from Sigma-Aldrich (St. Louis, MO, USA). Dichloromethane (DCM), diethyl ether, ethyl acetate, hexane, acetonitrile (ACN), methanol (MeOH), optima-grade water, phosphate-buffered saline (PBS), sodium hydroxide, gentamicin, potassium phosphate monobasic (KH_2_PO_4_), Dulbecco’s Modified Eagle’s Medium (DMEM), hydrochloric acid (HCl), and formic acid (FA) were purchased from Thermo Fisher Scientific (Waltham, MA, USA). Cell culture-grade water (endotoxin-free) was purchased from Cytiva (Logan, UT, USA). 

### 2.2. M2VON Synthesis and Characterization 

We synthesized the M2VON prodrug by esterifying the carboxylic functional group of VON, producing a lipophilic prodrug. Briefly, VON (1 equivalent, 2 g scale) was dissolved in a mixture of anhydrous DMF and chloroform (15 mL each). The solution was then cooled on a regular ice bath before adding 1.5 equivalents of HATU, 2 equivalents of triethyl amine, and 1.2 equivalents of 1-octadecanol. The reaction mixture (under an argon atmosphere) was then heated at 45 °C for 16 h under stirring. Upon completion, the mixture was cooled to room temperature and partitioned between dichloromethane and 0.1M HCl. The dichloromethane layer was washed with saturated sodium bicarbonate and brine. The organic extract was dried over sodium sulfate and concentrated by rotary evaporation. The dried sample was purified by silica column chromatography, eluting with a 6:1 hexane/ethyl acetate mixture, followed by a 4:1 hexane/ethyl acetate mixture. The desired compound fractions were dried on a rotary evaporator and placed under high vacuum for at least 16 h to obtain the M2VON prodrug as an off-white powder in >70% yield. The chemical structure and purity were confirmed by proton (^1^H) and carbon (^13^C) NMR spectroscopy on a Bruker Avance III HD 500 MHz spectrometer (Billerica, MA) at 11.7 T. The molecular mass ion of M2VON was confirmed with a peak at 743.65 *m*/*z* corresponding to the [M+H]^+^ adduct by infusing the prodrug on a Xevo TQ-XS Triple Quadrupole mass spectrometry system (Milford, MA, USA) operating in positive ion mode. 

### 2.3. NM2VON Nanosuspensions

Surfactant-stabilized aqueous nanosuspensions of NM2VON were developed using high-pressure homogenization. Briefly, the water-insoluble M2VON prodrug powder was pre-dispersed in a PBS buffer (PH = 7.044) solution of Tween-20 and PEG-3350 at ratios ranging from 20:1:1 to 7:1:1 (M2VON prodrug powder: Tween-20: PEG-3350). The microsuspension was then subjected to a nano-milling-compatible high-pressure homogenization process using an Avestin EmulsiFlex-C3 high-pressure homogenizer (Ottawa, ON, Canada) operated at pressures ranging from 15,000 to 20,000 PSI. The resultant nanosuspension was characterized for hydrodynamic particle size, polydispersity index (PDI), and zeta potential using a Malvern Zetasizer Nano-ZS (Westborough, MA, USA) equipped with Malvern Zetasizer Software v7.13. Samples were diluted 1:100 or 1:1000 in endotoxin-free water (Cytiva, Logan, UT, USA), depending on the formulation concentration. A 1 mL aliquot of the diluted sample was transferred into a BRANDTECH 1.5 mL semi-micro disposable cuvette (Essex, CT, USA) and inserted into the instrument with the triangle facing the front. Measurements were performed in manual mode under the following conditions: sample material set to polystyrene latex (refractive index = 1.590, absorption = 0.010); dispersant set to water (refractive index = 1.330, viscosity = 0.8872 cP) at 25.0 °C; equilibration time of 60 s; and a disposable cuvette (ZEN0040) selected as the cell type. Measurements were conducted at a backscatter detection angle of 173° (NIBS default) with an automatic measurement duration and three runs per sample. The drug content was quantified using UV-Vis UPLC analysis. Long-term formulation stability was evaluated by monitoring changes in prodrug content, particle size, PDI, and zeta potential under the storage conditions. M2VON purity and drug concentrations were quantified on a Waters Acquity H-Class UPLC system coupled with a TUV detector (Milford, MA, USA) and Waters Empower 3 software (Version 7.40.00.00). Separation was achieved on an Acquity UPLC BEH Shield RP18 column (1.7 µm, 2.1 × 30 mm) with detection at 280 nm wavelength. Isocratic elution was performed using a mobile phase consisting of 98% acetonitrile (ACN) with 0.1% formic acid and 2% water with 0.1% formic acid at a flow rate of 0.4 mL/min. The column temperature was maintained at 45 °C.

### 2.4. Cell Culture Treatment

For the in vitro HSC experiments, LX2 cells obtained from Sigma-Aldrich (St. Louis, MO, USA) were used. Cells were cultured according to the manufacturer’s protocol. They were treated with non-toxic concentrations of VON and NM2VON (100 µM) for 8 h and then activated with 10 ng/mL TGFβ. RNAs were extracted for RT-PCR, and cell lysates were collected for western blotting.

### 2.5. Cell Viability Assay

The cytotoxicity of VON and NM2VON was evaluated in human monocyte-derived macrophages (MDMs) using an MTT assay. MDMs were treated with increasing concentrations of VON or NM2VON (1.95–1000 µM) for 24 h under standard culture conditions. Vehicle-treated cells were used as controls. After treatment, cells were gently washed and incubated with MTT reagent in complete culture medium for 45 min. The medium was then removed, and 200 µL of dimethyl sulfoxide (DMSO) was added to each well to dissolve the formazan crystals. Absorbance was measured at 490 nm using a SpectraMax M3 microplate reader (Molecular Devices). Data were analyzed using SoftMax Pro software (version 6.2).

### 2.6. Animals

This study was approved by the Institutional Animal Care and Use Committee of the University of Nebraska Medical Center. For Animal Research, we followed the In Vivo Experiment reporting guidelines to prepare the data presented in this manuscript. We housed the animals under pathogen-free conditions and fed them a standard pelleted diet. We used seven-month-old non-transgenic C57BL/6 males (4 per group) and HBV transgenic (Tg05) mice (5 males and 5 females per group) expressing 1.3 HBV wild-type viral genomes. Transgenic animals were obtained from J.-H. James Ou, University of Southern California [11]. Male Sprague–Dawley (SD) rats were randomly assigned to three treatment groups (n = 3 per group). The animals were shaved at the injection site and received a single intramuscular injection of 300 μL NM2VON, corresponding to an average VON-equivalent dose of 189–200 mg/kg. The animals were euthanized on days 3, 14, and 28 post-administration. At each time point, an age- and sex-matched untreated control animal was concurrently euthanized with the treated animals. The animals were housed under pathogen-free conditions and were fed a standard diet.

### 2.7. VON Plasma and Tissue Quantitation 

VON concentrations in plasma and liver tissues were quantified using ultra-performance liquid chromatography–tandem mass spectrometry (UPLC–MS/MS). Analyses were performed using a Waters ACQUITY H-Class UPLC system coupled to an Xevo TQ-XS triple quadrupole mass spectrometer (Waters Corporation, Milford, MA, USA) operated in positive electrospray ionization (ESI^+^) mode. Data acquisition and quantification were conducted using MassLynx 4.2 with the TargetLynx XS software. Deuterated dolutegravir (DTG-d3) was used as an internal standard (IS) at a final concentration of 0.8 ng/mL. For liver analysis, the tissues were homogenized in four volumes of 90% methanol containing 0.1% formic acid. Plasma samples were processed directly, without homogenization. For extraction, 10 µL of plasma or liver homogenate was added to acetonitrile containing 0.1% formic acid and 10 µL of the DTG-d3 internal standard. The samples were vortex-mixed and centrifuged at 20,000× *g* for 10 min at 4 °C. Following centrifugation, 80 µL of the supernatant was transferred, diluted with water containing 0.1% formic acid, vortexed, and placed into autosampler vials. A 3 µL aliquot was injected for UPLC–MS/MS analysis. Calibration standards were prepared by spiking VON into control plasma or liver homogenate over a concentration range of 2–5000 ng/mL. Matrix-matched quality control (QC) samples at low, medium, and high concentrations were processed in parallel with the study samples. The total matrix dilution factor was 100×. 

Chromatographic separation was achieved using a Waters ACQUITY UPLC BEH C18 column (1.7 µm, 2.1 × 50 mm) maintained at 45 °C. Mobile phase A consisted of water with 0.1% formic acid, and mobile phase B consisted of acetonitrile with 0.1% of formic acid. The flow rate was 0.5 mL/min, and a 7-min gradient was employed: initial conditions at 20% B, increased to 55% B in 1.5 min, ramped to 95% B in 6.0 min, held briefly, and returned to initial conditions for column re-equilibration. VON was monitored using the MRM transition *m*/*z* 490.83 → 216.02, while DTG-d3 was monitored using *m*/*z* 422.97 → 280.07. Additional qualifier transitions for VON included *m*/*z* 490.83 → 232.12, 490.83 → 237.98, and 490.83 → 279.97, respectively. The cone voltage for VON was set to 26 V, with collision energies ranging from 18 to 76 eV, depending on the transition. DTG-d3 was detected at a cone voltage of 22 V and collision energies of 24–52 eV. The retention times for DTG-d3 and VON were approximately 2.33 min, and 3.43 min. Quantification was based on the peak area ratio of VON to DTG-d3, which was normalized to an internal standard. All runs met the predefined acceptance criteria for calibration curve linearity and QC accuracy.

For toxicology and histological evaluations, whole blood was collected at necropsy and analyzed for routine hematological parameters using an Abaxis VetScan HM5 hematology analyzer. For serum chemistry analysis, blood samples were centrifuged at 2000× *g* for 8 min to isolate the serum, which was subsequently analyzed using an Abaxis VetScan VS2 analyzer. The collected tissues were fixed overnight in 4% paraformaldehyde, dehydrated through graded alcohol, embedded in paraffin, and sectioned at 5 μm. Tissue sections were stained with hematoxylin and eosin for histopathological evaluation. Images were acquired using a Nikon Eclipse E600 microscope, equipped with an Infinity 2 camera. Histopathological evaluation was performed in a blinded manner.

### 2.8. NM2VON Administration

Mice were injected with 1 μg/g body weight of CCl_4_ in corn oil twice a week using a 27-gauge needle, as described previously [12]. We performed the first intramuscular (IM) injection of NM2VON 5 days before the first CCl_4_ injection and repeated it on days 31 and 62. The animals were euthanized 24 h after the last CCl_4_ injection. 

### 2.9. RT-PCR Analysis for Pro-Fibrotic Markers 

Reagents for RNA and DNA isolation, cDNA synthesis, and RT-PCR were obtained from Life Technologies (Carlsbad, CA, USA). Total RNA was isolated from cells using TRIzol reagent. A two-step procedure was applied, in which 400 ng of RNA was reverse-transcribed to cDNA using the High-Capacity Reverse Transcription Kit. The cDNA was then amplified using TaqMan Universal Master Mix II with fluorescent-labelled primers (TaqMan Gene Expression Systems). After incubation in a model 7500 quantitative RT-PCR thermal cycler, the relative quantity of each RNA transcript was calculated using its threshold cycle (Ct) after subtracting that of the reference cDNA (GAPDH). Data were expressed as transcript quantities using relative quantification (RQ).

### 2.10. Western Blots and Antibodies

Cell samples were lysed in radioimmunoprecipitation assay buffer containing freshly added EDTA-free protease and phosphatase inhibitor tablets for 30 min on ice. The protein samples were resolved on a 10% SDS-PAGE gel, transferred to a nitrocellulose membrane, and blocked with 5% BSA for 1 h at RT. The membranes were incubated with primary antibodies, including GLI1 monoclonal antibody (Proteintech, 66905-1-Ig), monoclonal patched/PTCH1 Antibody (H-10) (Santa Cruz Biotechnology, sc-518102), monoclonal SMO/Smoothened antibody (E-5) (Santa Cruz Biotechnology, sc-166685), and GAPDH monoclonal antibody (Proteintech, 60004-1-Ig). After secondary antibody incubation at RT for 1 h, the membranes were developed using an enhanced chemiluminescent reagent. Images were captured using an iBright 750 imaging system and quantified using the Image J analysis software.

### 2.11. Quantification of HBV DNA and Metabolic Tests

HBV DNA levels were measured in the peripheral blood using the COBAS TaqMan HBV Test (Roche Diagnostics, Basel, Switzerland). The lower detection limit was 20 IU/mL (1 IU = 5.6 DNA copies). The samples were diluted 17.5-fold, and the detection limit was 350 IU/mL. Serum ALT and aspartate aminotransferase (AST) levels were determined using an automatic blood biochemical analyzer (Beckman Coulter LX20, Beckman Coulter, Brea, CA, USA).

### 2.12. TGFβ and Protein Kinase C (PKC) Activity

The whole lung was homogenized in ice-cold PBS and centrifuged at 2150× *g* for 10 min at 4 °C. The supernatants were decanted, and the total protein was assayed [13]. TGFβ1 was measured using a sandwich ELISA kit (ab119557; Abcam, Cambridge, UK), as previously described [14]. Whole lung homogenates were centrifuged at 4 °C for 30 min at 10,000× *g*. The supernatant (cytosolic fraction) was removed, and the pellet (particulate fraction) was resuspended in cell lysis buffer containing Triton X-100 (0.01%) and sonicated again. PKC activity was determined in crude whole-cell cytosolic and particulate fractions, as described previously [15]. A reaction mix was prepared with phorbol myristate acetate (PMA; 24 μg/mL), dithiothreitol (30 mM), ATP (150 μM), Mg-acetate (45 mM), PKC-specific substrate peptide (Calbiochem), and 10 μCi/mL [gamma-^32^P]-ATP were mixed in a Tris-HCl buffer (pH 7.5). Cytosolic or particulate samples (20 μL) were added to 40 μL of the reaction mix and incubated for 15 min at 30 °C. The reaction was halted by spotting 60 μL onto phosphocellulose exchange plates (St. Vincent’s Institute of Medical Research, Fitzroy, Australia). The plates were washed five times, dried, and counted in a non-aqueous scintillant (National Diagnostics, Atlanta, GA, USA). PKC activity was expressed as picomoles of phosphate incorporated per minute per milligram of cellular protein.

### 2.13. Lung Histology

Tissues were fixed with 4% paraformaldehyde overnight at 4 °C and embedded in paraffin. Five-micrometer sections were cut from the paraffin blocks, mounted on glass slides, and subjected to immunohistochemical staining using rabbit monoclonal αSMA antibodies (Thermo Fisher Scientific, Waltham, MA, USA). Polymer-based horseradish peroxidase–conjugated anti-mouse systems were used as secondary detection reagents, and the reaction was developed with 3,3′-diaminobenzidine. All paraffin-embedded sections were counterstained with Mayer hematoxylin. Bright-field images were obtained using a Leica DM1000 light-emitting diode under original magnification of ×100. Connective tissue was stained with picro-sirius red using a Picro-Sirius Red Stain kit (Abcam, ab150681) according to the manufacturer’s instructions. Mouse lung tissues obtained from the two treatment groups were excised, inflated to 15 cm H_2_O pressure with 10% formalin (Fisher Scientific, Fair Lawn, NJ, USA) for 24 h to preserve pulmonary architecture, and sectioned (5 mm) for histochemical analysis with Hematoxylin and Eosin (H&E) and Masson’s trichrome staining. For the visualization of inflammatory cell infiltration and signs of lung fibrosis, sectioned lungs were semi-quantitatively assessed in a blinded manner for the degree and distribution of lung inflammation using a previously published scoring system [16]. This scoring system evaluates the spectrum of inflammatory changes in the alveolar and bronchiolar compartments, assigning each parameter a value from 0 to 8, with higher scores indicating greater inflammation and fibrosis. Stained sections were examined using light microscopy (Zeiss Primo Star; Oberkochen, Germany), and images were captured using a digital camera (Canon, Melville, NY, USA).

### 2.14. Statistical Analysis 

Statistical significance was determined using GraphPad Prism version 10 by one-way and two-way analyses of variance and *t*-tests, with *p* ≤ 0.05 being considered significant. The experimental details are provided in the figure legends.

## 3. Results

### 3.1. Preparation and Physicochemical Characterization of M2VON

**Figure 1:**
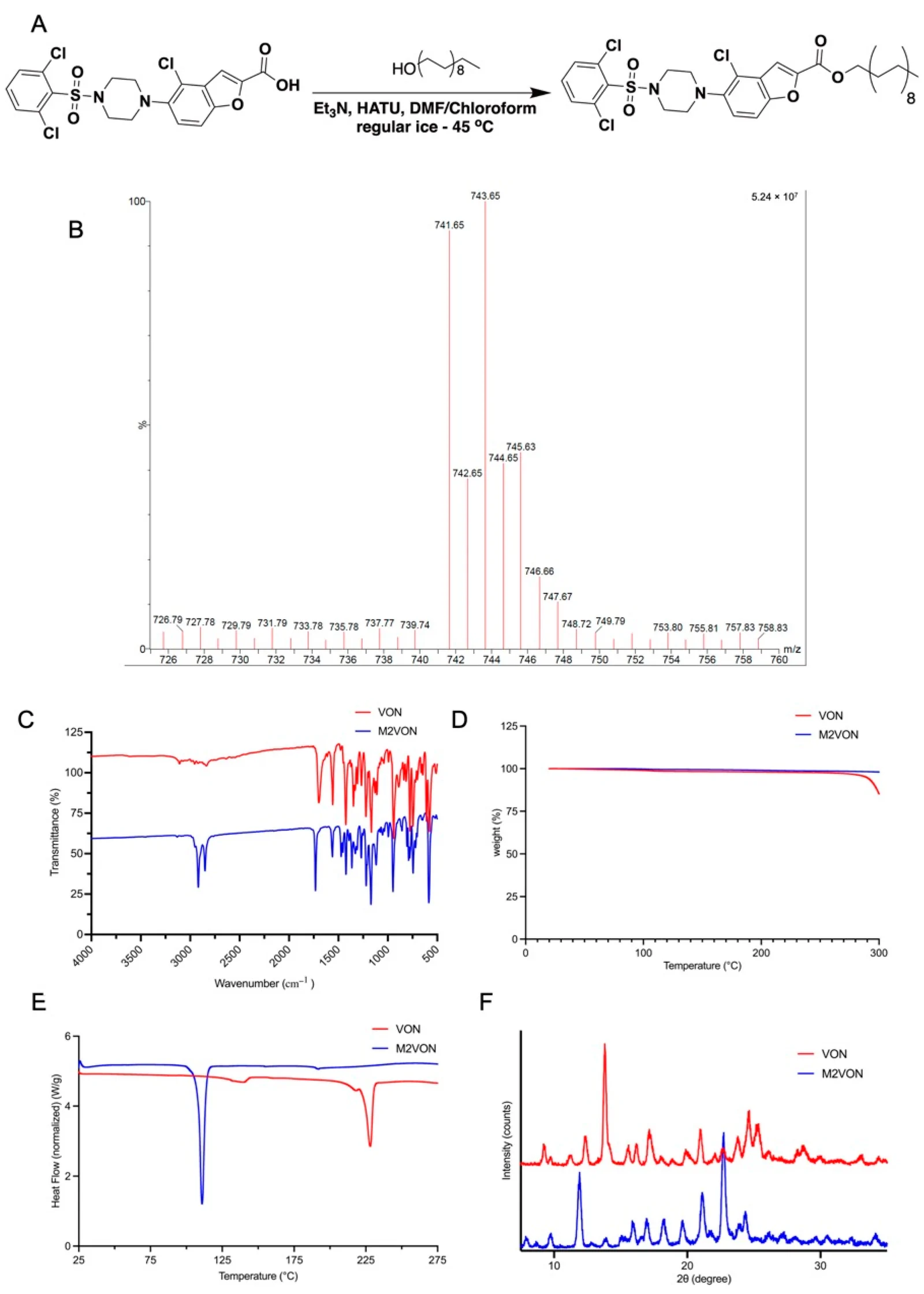
Synthesis and characterization of M2VON. (**A**) VON was chemically modified to M2VON through a one-step esterification reaction. (**B**) ESI infusion of M2VON on a Xevo TQ-XS Triple Quadrupole mass spectrometer produced a strong monoisotopic signal at 743.65 *m*/*z*, confirming the desired molecular weight. (**C**) FT-IR spectra overlay of Von (red) and M2VON (blue) showing characteristic absorption bands at 2920 cm^−1^ and 2846 cm^−1^ (C–H stretching of the long alkyl chain) and 1732 cm^−1^ (C=O ester stretches), confirming the successful prodrug formation. (**D**) TGA profiles of Von and M2VON, collected at a ramp rate of 10 °C/min from 30 °C to 600 °C, confirmed the anhydrous nature and thermal stability of the two compounds. (**E**) DSC thermograms revealed distinct endotherms for M2VON compared to Von, with the prodrug exhibiting a lower melting point owing to lipid conjugation. (**F**) XRD patterns (2θ range: 2–70°, scan rate: 1°/min) identified the differences in the crystalline forms of the parent drug and prodrug.

Lipophilic injectable prodrug formulations have demonstrated potential for enhancing drug half-life and enabling targeted tissue delivery [17]. The less water-soluble prodrug formulation forms a prolonged drug depot at the injection site, resulting in absorption-rate–limited (flip-flop) pharmacokinetics (PK). The extension of drug half-life is further governed by a dynamic interplay among prodrug hydrophobicity and lipophilicity, rates of chemical and enzymatic processes, and sequestration in histocytes at the injection site and in peripheral lymphoid tissues. Importantly, prodrug design serves to preclude adverse reactions to the parent drug. In this study, M2VON, an ester prodrug, was synthesized using a one-step, scalable process (Figure 1A). The selection of the stearyl alcohol prodrug moiety was based on the established safety profile of the lipid and prior work from our laboratory, where stearoylated antiviral agents exhibited optimal PK and safety profiles compared to other prodrug linkages [18,19,20,21].

The prodrug breakdown product, stearoyl alcohol, is safe because it is a common food additive. The chemical synthesis process produced the target compound with an 80% chemical yield, high purity, and reproducibility. The chemical structure of M2VON was confirmed by ^1^H and ^13^C Nuclear Magnetic Resonance (NMR) spectroscopy, mass spectrometry infusion, and Fourier-Transform Infrared Spectroscopy (FT-IR) (Figure 1B,C and Supplementary Figures S1 and S2). The triplet at 0.87 ppm and the broad peak at 1.27 ppm in the prodrug ^1^H NMR spectrum represent the terminal methyl and methylene protons of the lipid conjugate (Supplementary Figure S1). Mass spectrometry of M2VON produced a strong monoisotopic peak at 743.65, confirming the desired molecular weight (Figure 1B). The FT-IR spectrum exhibited characteristic absorption bands at 2920 cm^−1^ and 2846 cm^−1^ (C–H stretching of aliphatic chains) and 1732 cm^−1^ (C=O stretching of the ester), consistent with the expected chemical structure (Figure 1C). Thermogravimetric analysis (TGA) further confirmed the anhydrous nature of the M2VON powder over 0–390 °C (Figure 1D). Differential Scanning Calorimetry (DSC) thermograms of M2VON showed distinct thermal transitions, with a lower melting point for M2VON, confirming the attachment of a lower-melting-point lipid to VON (Figure 1E). X-ray diffraction (XRD) analysis showed that M2VON exhibited a distinct crystalline pattern compared with the parent compound (Figure 1F). Importantly, this well-defined crystalline pattern supports the development of stable nanosuspensions with predictable PK profiles.

### 3.2. M2VON Anti-Fibrotic Activities

**Figure 2:**
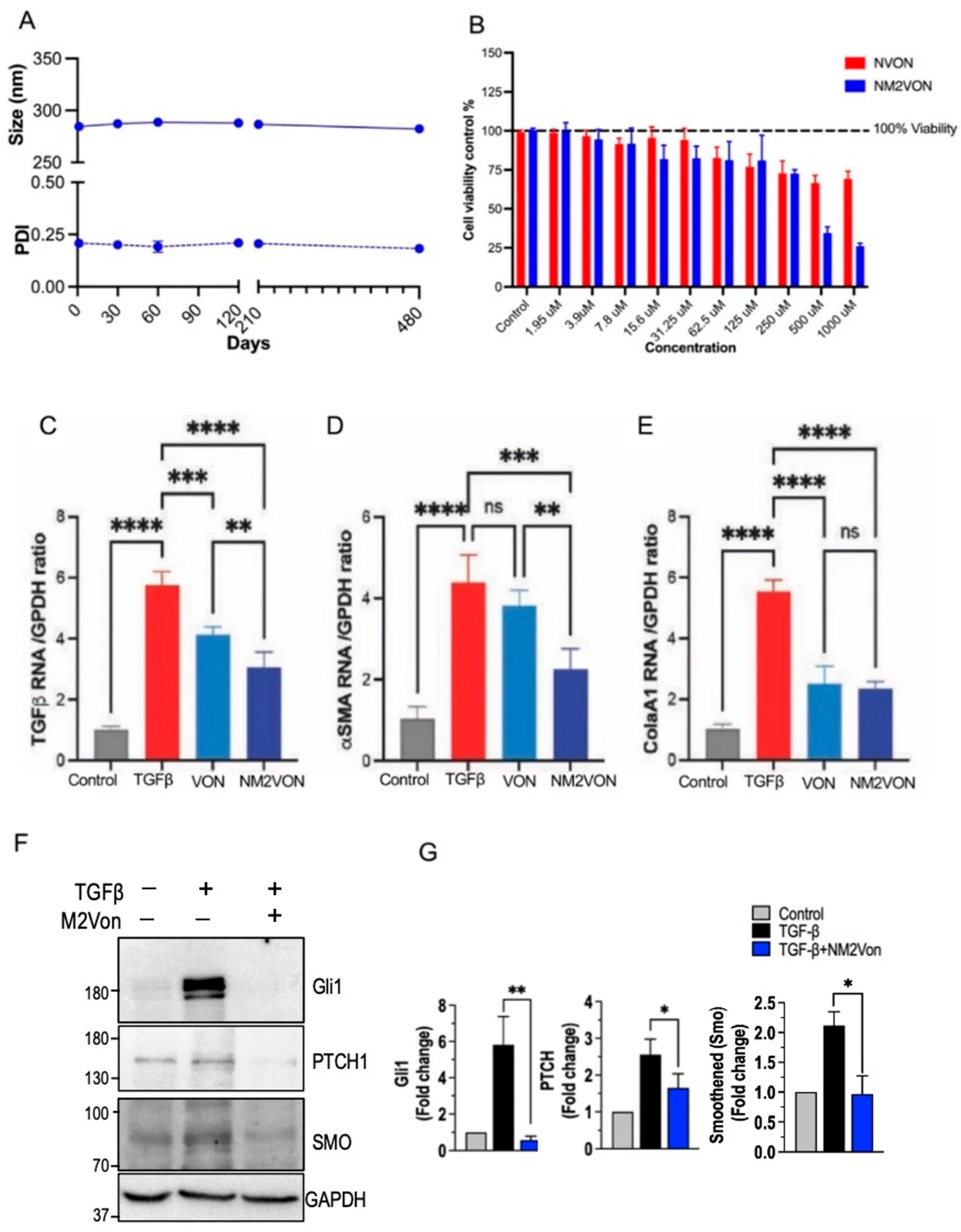
NM2VON characterization and in vitro anti-fibrotic efficacy evaluation (**A**) Long-term stability of the NM2VON nanosuspension over 480 days was assessed using dynamic light scattering, which showed consistent particle size (green) and PDI (magenta), confirming the formulation stability during long-term storage. (**B**) In vitro cytotoxicity of VON (red) and NM2VON (blue) was evaluated in cultured human MDMs after 24 h of exposure to a concentration range of 1.95–1000 µM using a standard cell viability assay. Data are presented as means ± SEM, N = 3 independent replicates. All experiments were repeated at least thrice, yielding reproducible results. LX2 cells were incubated in 2% FBS with no starvation. The concentration of Von and NM2Von used in the experiments shown in Figure 2 (**C**–**G**) was 100 µM. We added Von and NM2Von for 8 h, washed them out, then applied TGFβ (10 ng/mL) for 48 h. The expression of activation markers (**C**) TGFb, (**D**) a-SMA, and (**E**) ColaA1 were assessed by RT-PCR 48 h later. One-way ANOVA revealed a significant reduction, and NM2VON effects exceeded those of the parent drug for TGFβ and αSMA. **—*p* < 0.001, ***—*p* < 0.001, ****—*p* < 0.0001. (**F**) Representative image showing the effect of NM2Von on Hh proteins. LX2 cells were seeded at a density of 400,000 cells/well. Cells were treated with NM2VON (100 µM) for 8 h, followed by the addition of TGFβ (10 ng/mL) as described above. After a 48-h incubation period, cells were harvested for protein analysis by western blotting (WB). (**G**) WB quantification is based on three independent experiments and is shown as the fold response relative to the control, TGFβ, and TGFβ + NM2VON-treated samples. One-way ANOVA revealed a significant reduction: * *p* < 0.01, ** *p* < 0.001. Original WB images can be found in Supplementary Figure S8.

Several aqueous-based LA injectables have improved the treatment and prevention outcomes of chronic diseases [22,23]. Following administration, the prodrug nanocrystals are taken up by histocytes, enabling gradual intracellular dissolution and prodrug activation [18]. Together with cellular sequestration, the rate of drug absorption and dissolution underpins a prolonged drug half-life and action. A readily scalable high-pressure homogenization process was used to manufacture NM2VON formulations in an aqueous buffer containing polyethylene glycol and polysorbate-20 (Tween 20) as stabilizing surfactants. NM2VON formulations were produced with a drug loading of >78%. The average particle size and polydispersity index (PDI) of NM2VON were 280 ± 2.5 nm and 0.21 ± 0.02, respectively, indicating a homogeneous suspension (Figure 2A). The formulation was readily syringeable through a 25-G needle, confirming its low viscosity. The formulation remained stable for up to 480 days, with no significant changes in particle size, prodrug concentration or uniformity. On day 480, the particle size and PDI were 282 ± 1.3 nm and 0.18 ± 0.03, respectively (Figure 2A). The safety profile of NM2VON in monocyte-derived macrophages (MDM) was evaluated across a concentration range of 1.95–1000 µM using the MTT assay. Both NM2VON and NVON exhibited comparable effect on cell viability at concentrations of ≤250 µM (Figure 2B). 

The anti-fibrotic effects of NM2VON were evaluated in LX-2 cells, an immortalized HSC cell line. HSC activation is a central pathological event in liver fibrosis, characterized by transdifferentiation into myofibroblast-like cells that drive ECM deposition. LX2 cells were pretreated with either VON or NM2VON for 8 h, followed by stimulation with TGFβ for 48 h to induce fibrotic activation. αSMA, Col1A1, and TGFβ were quantified as established markers of hepatic fibrogenesis, reflecting HSC activation, ECM deposition, and profibrotic signaling. As expected, TGFβ stimulation robustly induced profibrotic gene expression in the untreated cells. Notably, NM2VON treatment conferred significantly greater protection against TGFβ-induced activation of LX2 cells compared to the parent drug VON, as demonstrated by the marked suppression of TGFβ, αSMA, and Col1A1 mRNA expressions (Figure 2C–E). These findings suggest enhanced intracellular delivery for the lipophilic NM2VON compared to VON since prodrugs are not pharmacologically active compounds.

To further elucidate the mechanism underlying NM2VON-mediated suppression of HSC activation, we examined its effects on the Hedgehog (Hh) signaling pathway, which has been implicated in TGFβ-driven fibrogenesis and HSC activation through the regulation of the transcription factor Gli2 [24]. The protein expression levels of key Hh pathway components, including Gli1, Patched (PTCH), and Smoothened (SMO), were assessed using western blot analysis in LX2 cell lysates following TGFβ activation with or without NM2VON treatment. Consistent with the observed anti-fibrotic effects, NM2VON markedly reduced the expression of Hh pathway proteins in TGFβ-activated HSCs compared to the controls. Representative immunoblots and fold-changes illustrating the suppression of Gli1, PTCH, and SMO are shown in Figure 2F and G, respectively.

### 3.3. NM2VON Reduced Fibrotic Changes in CCl_4_-Treated C57Bl/6 Mice 

**Figure 3:**
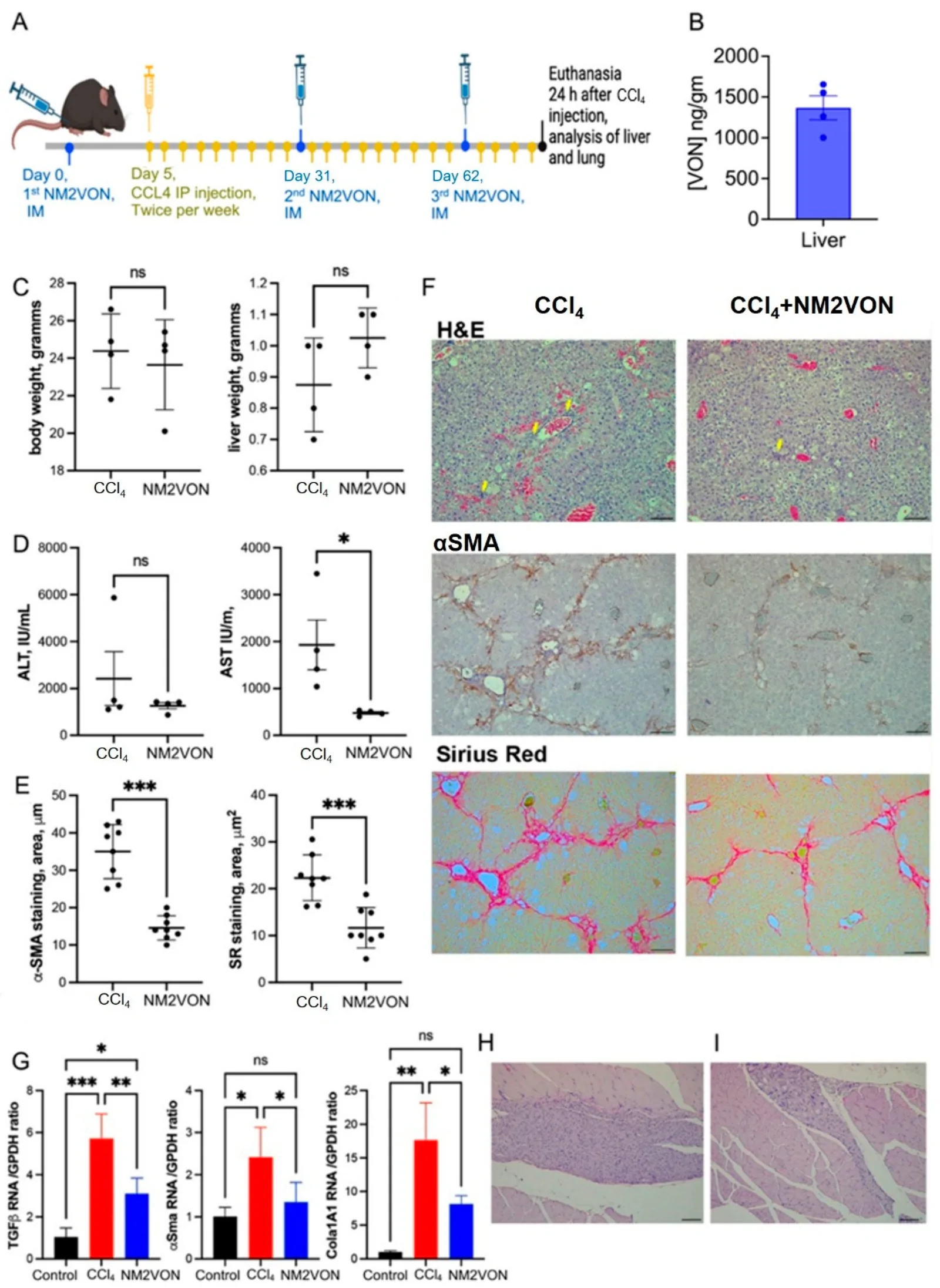
Monthly administration of NM2VON attenuated fibrosis development during chronic CCl_4_ administration. (**A**) Schematic of experimental design. C57BL/6 male mice received the first NM2VON IM injection at 200 mg/kg, and five days later received twice-weekly IP injections of 1 μg/g body weight of CCl4 in corn oil. Subsequent NM2VON IM doses of 200 mg/kg were administered in weeks 5 and 10. CCl_4_ injections were continued until week 12, followed by animal sacrifice 24 h after the final CCl_4_ injection. Blood samples, livers, lungs, kidneys, and injection sites were collected for biochemical, immunohistological, and RT-PCR analyses. (**B**) VON concentrations were quantified in liver tissues at study termination. Individual animal values are shown as mean ± SEM (n = 4). (**C**) Body and liver weights were similar between the CCl_4_ control and NM2VON-treated groups. (**D**) NM2VON treatment decreased AST levels. (**E**) Sirius red and αSMA staining areas were smaller in treated animals, and representative histological images are shown in (**F**). The scale bar is 100 mm. H&E staining showed reduced neutrophil infiltration (yellow arrows); (**G**) The mRNA expression levels of TGFβ, αSMA, and Col1A1 were reduced in mice treated with NM2VON. (**H**,**I**) The injection-site depot of NM2VON at 2 weeks (**H**) and 4 weeks (**I**) showed macrophage infiltration within the long-lasting formulation depot. *—*p* < 0.05, **—*p* < 0.01, ***—*p* < 0.001.

To evaluate the in vivo anti-fibrotic effect of NM2VON, liver fibrosis was induced in mice by chronic administration of CCl_4_, a model that recapitulates hepatotoxic injury–driven stellate cell activation and progressive ECM deposition [12]. Seven-month-old C57Bl/6 male mice were administered the first IM dose of NM2VON at a VON-equivalent dose of 200 mg/kg body weight. Five days later, the animals received intraperitoneal (IP) injections of CCl4 at 1 µg/g body weight twice weekly in corn oil. The second and third IM injections of NM2VON at a VON-equivalent dose of 200 mg/kg were administered at weeks 5 and 10. CCl_4_ injections were continued until week 12, after which the animals were euthanized 24 h after the last CCl_4_ administration (Figure 3A). At study termination, liver drug concentrations were 1455 ng/g tissue (Figure 3B), indicating drug-mediated efficacy. The NM2VON injections did not cause injection-site injuries. Animal and liver weights were comparable to those of age-matched controls, demonstrating the safety profile of repeated NM2VON dosing (Figure 3C). Longitudinal monitoring of body weight throughout the 12-week study similarly showed no treatment-related effects on the animal weight or overall health (Supplementary Figure S3). NM2VON treatment also improved liver injury, as demonstrated by the reduced ALT and AST levels. Blood AST levels measured at study termination were significantly lower, whereas ALT levels were not (Figure 3D). 

The reduction in collagen deposition was accompanied by a corresponding decrease in αSMA–positive staining, indicating the suppression of HSC activation. Representative liver sections confirmed diminished fibrotic architecture, reduced αSMA immunoreactivity, and decreased inflammatory cell infiltration, including fewer neutrophils, in NM2VON-treated animals compared to CCl_4_ control animals (Figure 3E). To evaluate whether sustained tissue drug delivery translated into protection against toxin-induced liver fibrosis, we assessed the expression levels of TGFβ, α-SMA, and Col1A1, pro-fibrotic markers, using RT-PCR and histology. Histochemical assessment showed marked anti-fibrotic activity, with Sirius Red staining revealing reduced collagen-positive areas in NM2VON-treated mice compared with untreated CCl_4_ controls (Figure 3F).

NM2VON treatment significantly downregulated key profibrotic markers in liver tissue. The histological findings were further supported by semiquantitative analysis of profibrogenic mRNA expression. Notably, hepatic TGFβ expression was reduced by approximately 1.5-fold in NM2VON-treated mice compared to untreated CCl_4_ mice, indicating effective suppression of central fibrogenic signaling. αSMA and Col1A1 mRNA expression was also significantly decreased by approximately 1.2-fold and 2-fold, respectively, consistent with reduced HSC activation and ECM production (Figure 3G).

H&E staining of the muscle injection site at weeks 2 and 4 after NM2VON dosing demonstrated an expected local inflammatory reaction to the drug depot, characterized by histiocytic cell infiltration, with variable amounts of lymphocytes, endothelial cells and fibroblasts (Figure 3H,I). Such local reactions to drug depots have been extensively described for other similar clinically approved long-acting injectable suspensions and are considered normal biological processes that facilitate drug absorption. Muscle tissue necrosis was recorded when injections reached the muscle fibers directly. 

### 3.4. NM2VON Controls Liver Fibrosis in HBV Transgenic Mice

Chronic HBV can lead to liver fibrosis, which can be attenuated by VON administration. These drug-induced antiviral activities were controlled in a phase 1b clinical trial [9]. To confirm and extend these observations, we evaluated the anti-fibrotic effect of LA NM2VON in HBV-transgenic mice. We administered CCl_4_ to the mice to induce liver fibrosis. The experimental design was consistent with that of control non-transgenic C57BL/6 mice (Figure 3A). We used equal numbers of male and female animals to assess sex-dependent differences in NM2VON therapeutic efficacy. 

**Figure 4:**
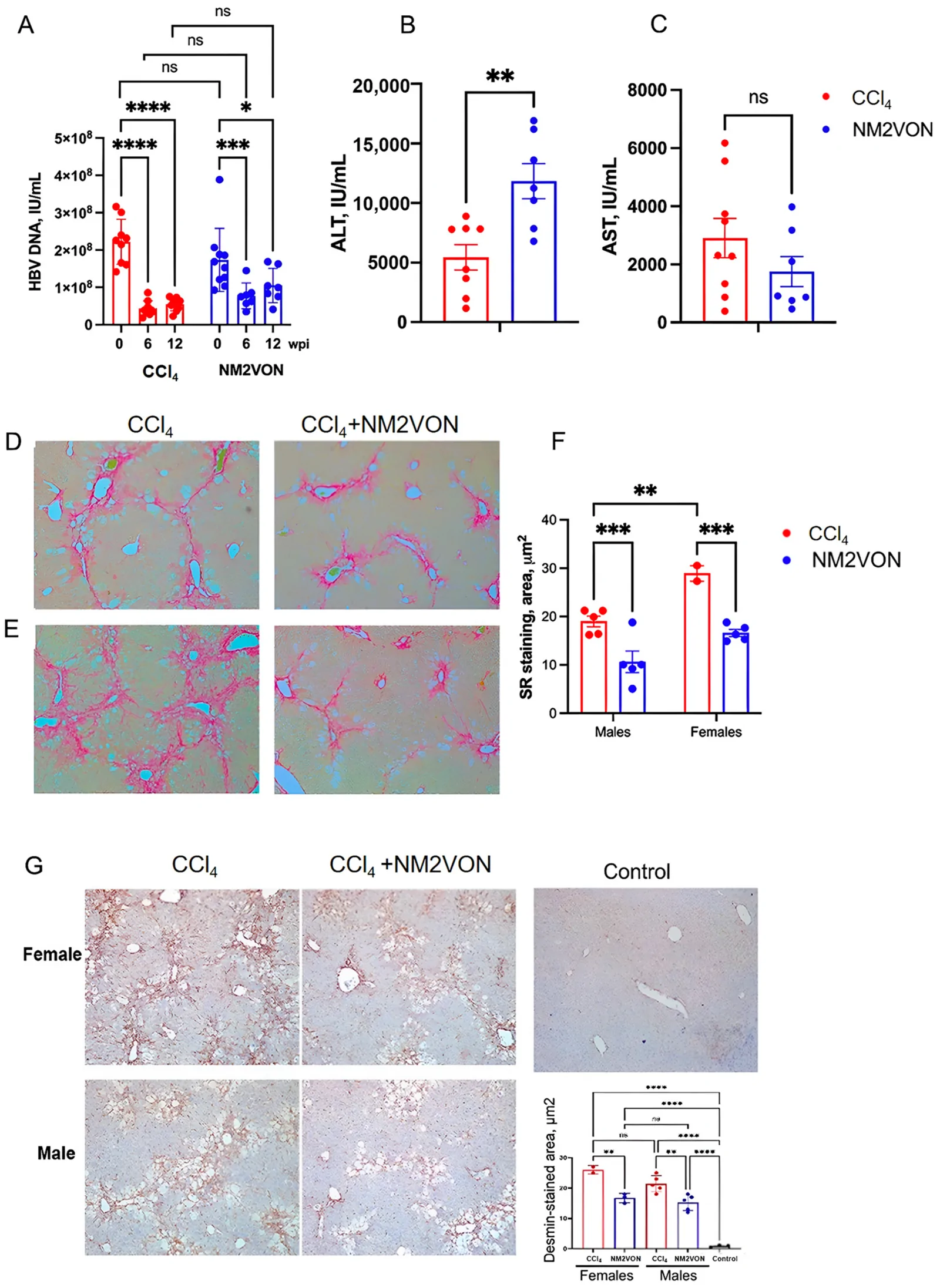
Effects of NM2VON on CCl_4_ treated HBV transgenic mice. HBV transgenic mice (male and female) were administered the first IM injection of NM2VON at 200 mg/kg. After 5 days, biweekly IP injections of CCl_4_ (1 µg/g body weight in corn oil) were administered. The second dose of MN2VON was administered in week 5 and the third at week 10. CCl_4_ injections continued until week 12, and the animals were euthanized 24 h after the last CCl_4_ dose. (**A**) HBV DNA, (**B**) ALT, and (**C**) AST levels in plasma. Individual values for CCl_4_-treated (red) and CCl_4_+NM2VON-treated (blue) mice, with means and SEM are shown. *—*p* < 0.05, **—*p* < 0.01, ***—*p* < 0.001, ****—*p* < 0.0001. Histological staining of liver tissues with Sirius Red (SR) in (**D**) male and (**E**) female mice. (**F**) Statistical evaluation of the stained areas showed increased fibrotic changes in females compared to males, as well as the anti-fibrotic effect of NM2VON treatment. Individual mouse values, means, and SEM are shown in the figure. **—*p* < 0.01, ***—*p* < 0.001. (**G**) Liver tissues of control, CCl_4_-administered female and male mice, as well as NM2VON+CCl_4_-treated mice, were stained with anti-Desmin antibodies. **—*p* < 0.01, ****—*p* < 0.0001.

Chronic CCl_4_ administration suppressed viral replication in HBV-infected transgenic mice, and NM2VON did not affect this effect (Figure 4A). Liver injury markers demonstrated modest changes in serum transaminase levels after treatment. ALT and AST levels were evaluated in plasma samples collected at the end of the study, with NM2VON-treated animals showing a trend toward reduced liver injury compared with CCl_4_ controls (Figure 4B,C). Notably, NM2VON significantly reduced fibrotic activation. Histological assessment using Sirius Red staining demonstrated robust anti-fibrotic activity, with a marked reduction in collagen deposition in the treated animals. There was 100% survival in NM2VON-treated mice compared to CCl_4_-only controls (Figure 4D,E). Quantitative analysis of stained liver sections confirmed a significantly lower fibrotic burden following NM2VON treatment, with female mice exhibiting greater baseline fibrosis than male mice (Figure 4F). Immunohistochemical staining for desmin, a marker of activated HSC, showed reduced desmin+ areas in NM2VON-treated male and female mice compared to the CCl_4_-only group (Figure 4G). No sex-dependent differences in desmin staining were observed in CCl_4_-exposed animals. Biochemical analysis of plasma samples collected 24 h after CCl_4_ injection revealed a modest reduction in AST levels in NM2VON-treated mice, whereas ALT levels were minimally elevated compared with the control group (Figure 4B). This may reflect the rate of liver regeneration, as the inhibition of hepatocyte division leads to the activation of ALT, which serves as a metabolic regulator to support liver recovery [25]. Notably, NM2VON protected all female mice from CCl_4_-induced mortality (100% survival) compared to CCl_4_-only females, in which three animals died.

**Figure 5:**
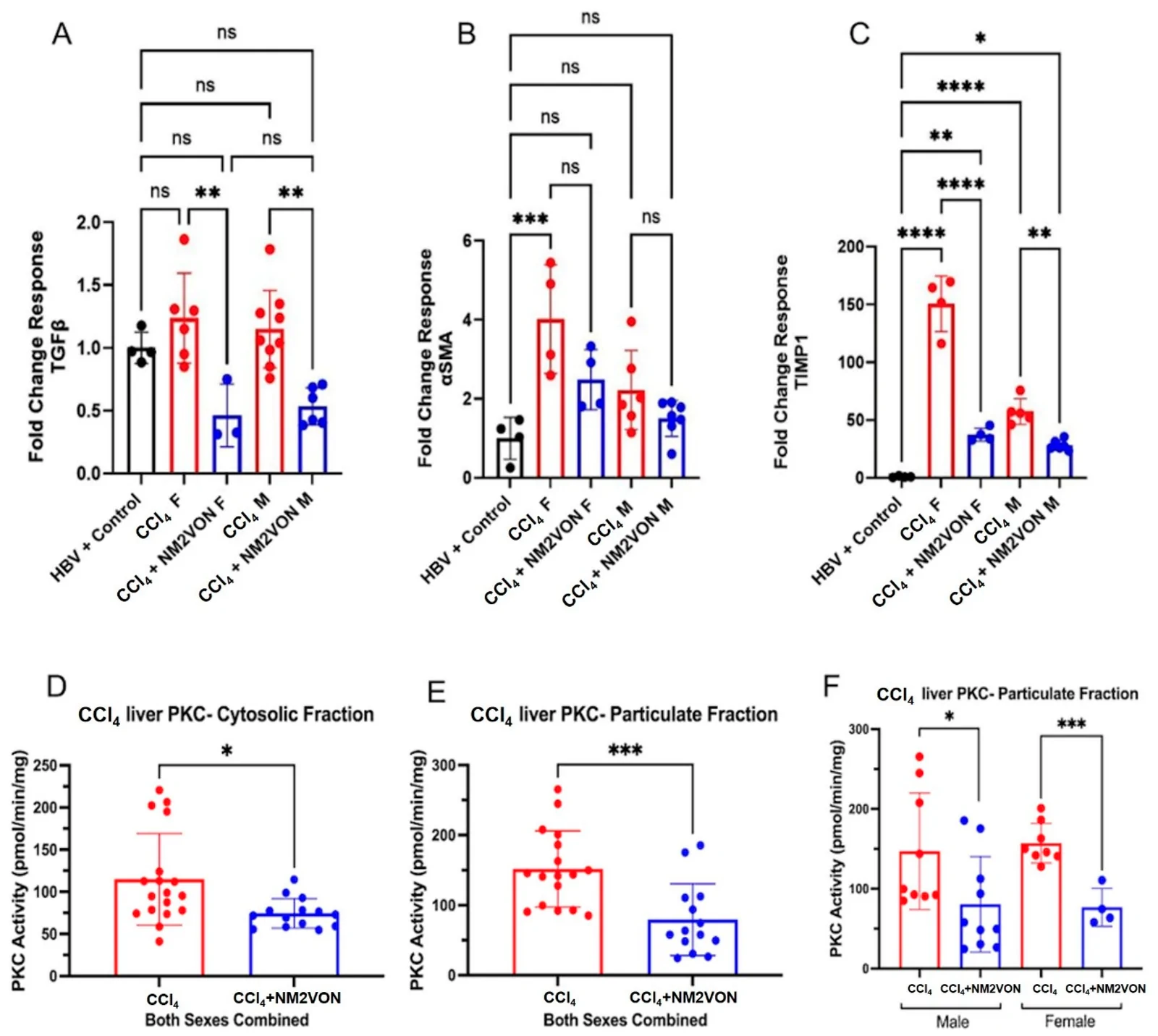
NM2VON downregulated CCl_4_-induced activation of pro-fibrotic mRNAs in the liver tissues of HBV-transgenic mice. Pro-fibrotic mRNAs, (**A**) TGFβ, (**B**) αSMA, and (**C**) TIMP1, were measured in the liver tissues of female and male mice. The results are expressed as fold responses. Individual values in female (F) vs. male (M) mice in CCl_4_- and NM2VON-treated mice, means, and SEM are shown. *—*p* < 0.05, **—*p* < 0.01, ***—*p* < 0.001, ****—*p* < 0.0001. Liver tissues were obtained from CCl_4_ and NM2VON- and NM2VON-treated mice and subdivided into cytosolic and particulate lipid-associated fractions for PKC activity assessment. (**D**) PKC activity in the cytosolic fraction of mouse liver. (**E**) PKC activity in the liver particulate fraction of the mouse liver. (**F**) PKC activity in the liver particulate fraction of male and female mice. The results are expressed as individual values, means, and SEM, *—*p* < 0.05, ***—*p* < 0.001.

To further validate the potential anti-fibrotic efficacy of NM2VON, we quantified hepatic mRNA expression of key profibrotic markers by RT-PCR (Figure 5A–C). As expected, chronic CCl_4_ administration significantly induced TGFβ mRNA expression in both male and female mice. The results reflected the activation of the fibrogenic signaling pathway. However, NM2VON treatment significantly suppressed this induction, resulting in markedly lower TGFβ expression than in CCl_4_-only controls in both sexes (Figure 5A). αSMA expression was also significantly elevated after CCl4 exposure, with a greater induction in female mice. NM2VON treatment reduced αSMA mRNA expression in both sexes, with a statistically significant reduction in females (Figure 5B), consistent with suppression of HSC activation. Tissue inhibitor of metalloproteinases 1 (TIMP1), a key regulator of ECM turnover and fibrosis stabilization, was strongly induced by CCl4 treatment. TIMP1 mRNA levels increased up to 150-fold in female mice and 50-fold in male mice compared to controls. These data underscore sex-dependent differences in fibrosis severity. Importantly, NM2VON administration significantly reduced TIMP1 expression in both male and female mice, indicating effective attenuation of matrix-stabilizing, profibrotic signaling, regardless of baseline induction (Figure 5C).

Given the central role of PKC signaling in regulating TGFβ activation and downstream fibrogenic pathways [26], we investigated whether NM2VON-mediated suppression of profibrotic gene expression was associated with altered hepatic PKC activity. We isolated cytosolic and particulate fractions from liver tissues of CCl4- and NM2VON-treated mice and quantified PKC activity. NM2VON treatment resulted in a significant reduction in PKC activity in both the cytosolic and particulate fractions compared to the CCl_4_-only controls (Figure 5D,E). This inhibitory effect was observed in both male and female mice, with no significant recorded sex differences for PKC suppression (Figure 5F). The coordinated reduction in TGFβ, αSMA, and TIMP1 expression, together with decreased PKC activity, provides mechanistic support for the antifibrotic effects observed in histological and biochemical examinations of NM2VON-treated animals.

### 3.5. NM2VON Reduced Lung Fibrosis in HBV Transgenic Mice

Given that chronic liver injury and systemic fibrogenic signaling can promote extrahepatic fibrosis, including pulmonary involvement, we evaluated whether CCl_4_ treatment induced lung fibrosis and whether NM2VON was protective. The study design was analogous to that shown in Figure 3A. Mouse lung tissues from the two treatment groups underwent histochemical analysis with H&E and Masson’s trichrome staining to assess tissue architecture, inflammatory infiltration, and collagen deposition. Fibrotic severity was quantified using Ashcroft scoring, and the anti-fibrotic effect of NM2VON was validated by measuring total lung TGFβ1 levels and PKC activity. 

**Figure 6:**
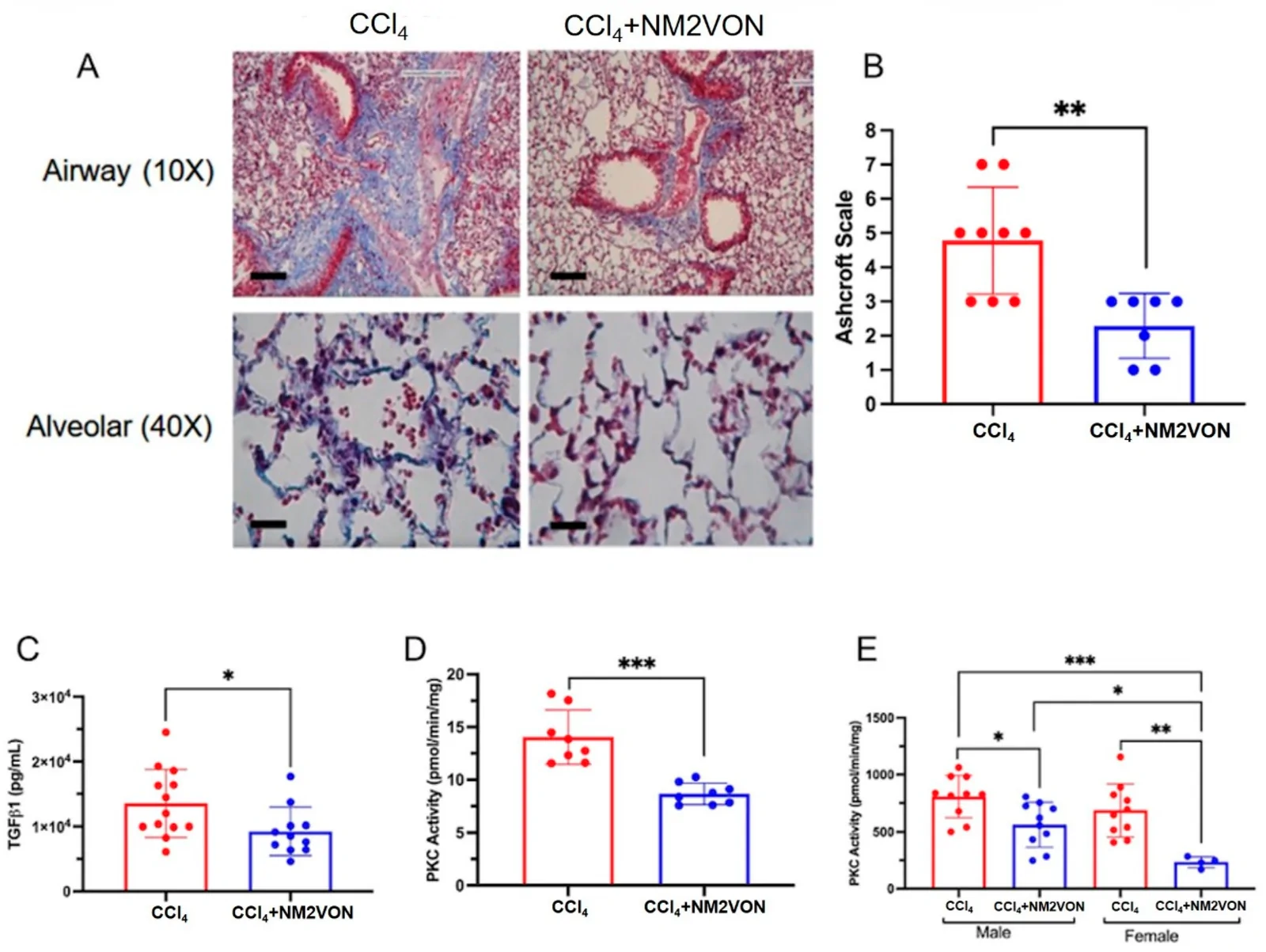
NM2VON attenuated fibrotic changes in both male and female transgenic HBV mice. (**A**) Representative Masson’s trichrome lung section images from both CCl_4_ treatment groups (Control and NM2VON) with representative images of collagen content (blue stain) shown for both proximal airway regions (10× magnification) and distal alveolar parenchyma (40×). The line scale was 100 μm and 25 µm. (**B**) Ashcroft Scoring with individual values averaged from 8 to 10 images per section per mouse. Statistical significance CCl_4_-treated vs. CCl_4_-treated with NM2VON (n = 7–9; *p* < 0.008). (**C**) ELISA of total lung homogenate TGFβ1 levels in mice treated with CCl_4_ in the presence or absence of NM2VON. (n = 11–13; *p* < 0.03). (**D**) PKC activity in total lung homogenates of CCl_4_-treated mice in the presence or absence of NM2VON, combined particulate fraction PKC activity; (n = 11–13; *p* < 0.0009) (**E**) Particulate fraction PKC activity in male vs. female mice. (n = 4–9; * *p* < 0.04, ** *p* < 0.001, *** *p* < 0.0001).

Histological examinations showed that control mice’s lungs preserved alveolar architecture. In contrast, lungs from CCl_4_-treated mice exhibited pronounced fibrotic remodeling, characterized by thickening of the lung parenchyma, distortion and disruption of alveolar structures, and prominent perivascular fibrosis (Figure 6A). Masson’s trichrome staining revealed extensive collagen accumulation in CCl_4_-exposed lungs, as evidenced by a marked increase in blue-stained collagen-rich areas (Figure 6A). Notably, monthly NM2VON treatment significantly attenuated fibrotic changes. Lung sections from NM2VON-treated mice showed a substantial reduction in collagen deposition compared to the CCl_4_ controls, with decreased collagen staining observed both in the perivascular regions and throughout the lung (Figure 6A). Quantitative assessment using Ashcroft scoring across all lung sections analyzed revealed a statistically significant reduction in overall lung fibrosis in NM2VON-treated animals compared to CCl_4_ control mice (Figure 6B).

Consistent with the histological data, analysis of lung sections showed a significant decrease in total lung TGFβ1 levels after NM2VON treatment, indicating suppression of profibrotic signaling in pulmonary tissue (Figure 6C). In parallel, PKC activity assessment showed that chronic CCl_4_ exposure predominantly enhanced PKC activation within the particulate (membrane-associated) fraction, whereas NM2VON treatment significantly reduced PKC activity in this fraction, indicating effective inhibition of CCl_4_-induced PKC signaling (Figure 6D). Although no sex differences in CCl4-mediated PKC activity were detected, NM2VON decreased PKC activity in both male and female mice, with a significantly greater NM2VON-induced decrease in females than in males (Figure 6E). These data support NM2VON anti-fibrotic activity.

### 3.6. NM2VON Injection Site Reactions and Drug Levels in SD Rats 

To further evaluate the tolerability of NM2VON, SD rats were administered a single intramuscular dose of 300 μL NM2VON (189–200 mg/kg NM2VON). Body weights were monitored over 28-day period, and the animals were euthanized on days 3, 14, and 28 for histopathological evaluation. Each time point included age- and sex-matched control animal groups. Complete hematologic and metabolic counts revealed no statistically significant differences between the treated and control groups, and the serum chemistry parameters remained comparable throughout the study (Supplementary Figure S4 and Table S1).

**Figure 7:**
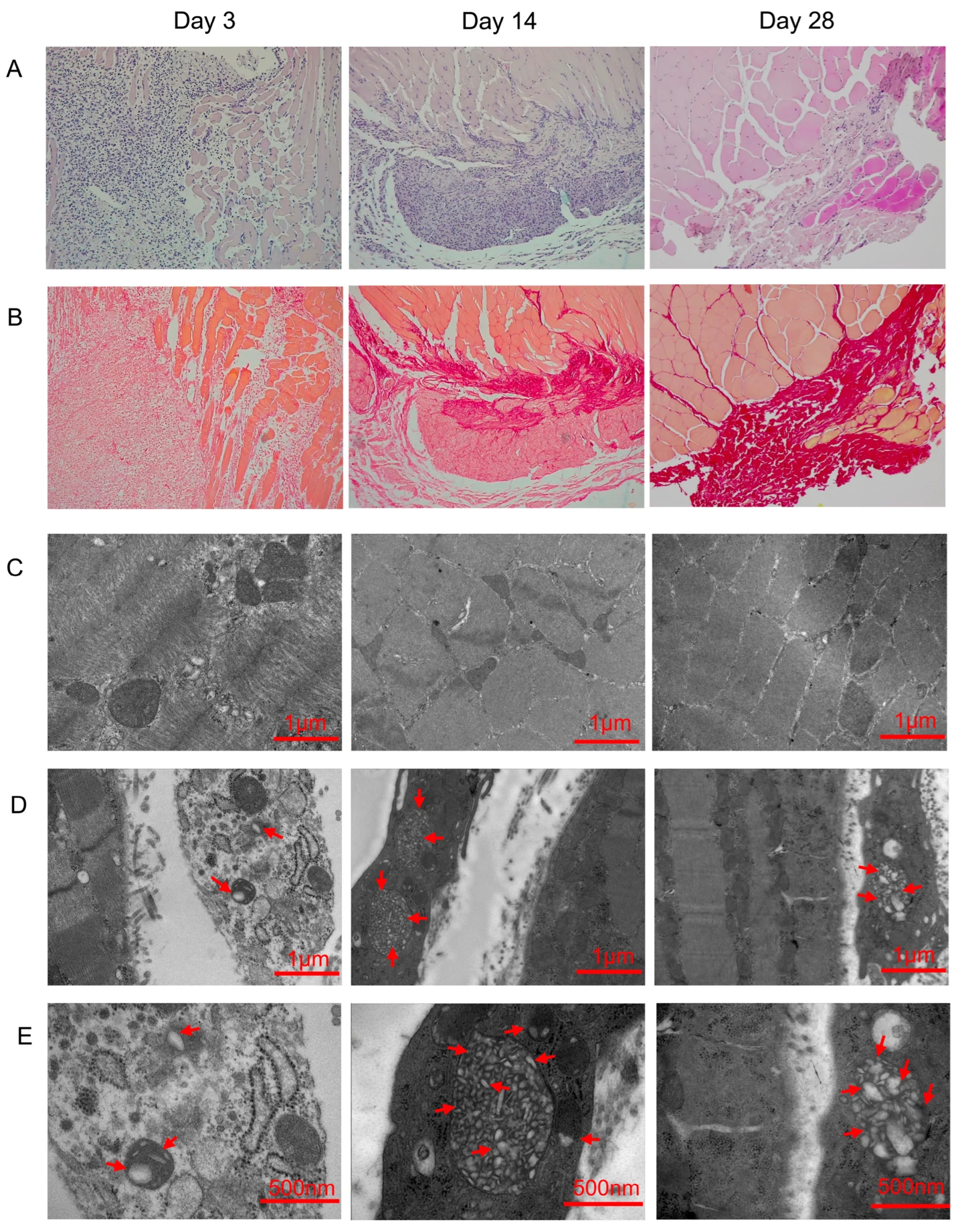
Histological and TEM characterization of the NM2VON nanosuspension at the injection site in SD rats. (**A**) H&E staining of muscle injection site tissues on days 3, 14, and 28 following intramuscular administration of NM2VON. The tissue sections showed localized histiocytic and inflammatory cell infiltration at the injection site, which persisted until day 28. (**B**) Picrosirius red–stained muscle sections from the injection site showing collagen deposition and tissue remodeling surrounding the NM2VON formulation depot over time. Representative images at 10× magnification are shown. (**C**) TEM images of control (uninjected) muscle tissue demonstrating normal ultrastructural morphology with no evidence of particulate material (scale bar = 1 µm). (**D**) TEM images of muscle tissue from NM2VON-treated animals showing infiltrating macrophages containing electron-dense NM2VON nanocrystals localized within intracellular vesicular compartments (scale bar = 1 µm). (**E**) Higher-magnification TEM images of NM2VON-treated muscle tissue demonstrating clusters of nanocrystals within endosomal/lysosomal vesicles of macrophages, consistent with phagocytic uptake and intracellular sequestration (scale bar = 500 nm). The red arrows indicate the nanocrystal-containing vesicles.

Histological evaluation of the muscle injection sites by H&E staining is shown in Figure 7A. Picrosirius red staining of muscle sections from the injection site (Figure 7B) demonstrated localized collagen deposition surrounding the formulation depot, indicative of tissue remodeling associated with a foreign-body granulomatous inflammatory response. The physiological response is expected for depot-based formulations and governs the rate of drug absorption. Collagen staining was most prominent at earlier time points and decreased over time, with no evidence of progressive fibrosis on day 28. Relative changes in body weight were similar between NM2VON-treated and control animals (Supplementary Figure S5) throughout the study. Muscle tissue at the injection site showed limited, localized reactions, with no evidence of myofiber degeneration after intramuscular NM2VON administration. The observed cellular responses were consistent with a transient foreign body reaction that was largely resolved by day 28, in accordance with the expected biological response to injectable depot-based formulations [27,28]. Examination of the liver, kidney, and spleen (Supplementary Figure S6) revealed no treatment-related or histopathological abnormalities.

Transmission electron microscopy (TEM) of injection-site tissues was used to further characterize cellular interactions. Muscle tissues from untreated control animals displayed normal ultrastructural morphology (Figure 7C). In contrast, tissues from NM2VON-treated animals demonstrated myeloid cell infiltration with clear evidence of nanoparticle uptake from days 3 to 28. Electron-dense clusters of nanocrystals were observed within the cytoplasm, predominantly localized to the endosomal vesicles (Figure 7D,E). Drug levels in the liver at the recorded time points were higher than plasma VON concentrations (Supplementary Figure S7). Importantly, no ultrastructural evidence of muscle degeneration was observed. Collectively, these findings indicate that intramuscular administration of NM2VON was well tolerated [27].

## 4. Discussion

In this study, we report the development and characterization of NM2VON, an LA prodrug formulation. Monthly NM2VON administration significantly attenuated toxin-induced fibrosis in a mouse model of liver damage. NM2VON treatment was associated with a marked reduction in collagen deposition, HSC activation, profibrotic gene expression, and fibrogenic pathways in liver and lung tissues. Reduced dosing intervals through rational prodrug design and subsequent formulation development highlight the therapeutic potential of LA FXR agonists for treating fibrosis. Importantly, in a randomized clinical trial of patients with fibrotic MASH, daily oral VON was safe and improved liver fat levels and renal function [7]. While these clinical findings are promising, long-term efficacy of daily oral VON in real-world settings may be compromised by longstanding non-adherence to frequently administered therapies. Several studies across other chronic disease areas suggest that LA treatments improve adherence, compliance, and patient satisfaction [29,30]. To this end, LA formulations for liver fibrosis are an emerging therapeutic approach that could revolutionize patient care. This was affirmed by the recent FDA approval of a once-a-week injectable Wegovy for MASH in patients with moderate-to-advanced fibrosis [31]. The development of longer-acting anti-fibrotic treatments could therefore potentially offer consistent and convenient options for patients seeking to sustain recovery without the burden of daily or weekly dosing schedules.

To convert VON into LA, we investigated a scalable lipophilic ester prodrug strategy. The ideal prodrug candidate should have optimal aqueous and octanol solubilities to support an aqueous-based formulation using a readily scalable milling process. The covalent linkage of stearyl alcohol to VON produced a crystalline water-insoluble prodrug with the desired physicochemical properties, enabling the production of surfactant-stabilized NM2VON nanocrystals. Notably, NM2VON nanocrystals remained stable for up to 480 days with no significant changes in particle size, homogeneity, or prodrug chemical stability. Importantly, the lipophilic nature of the M2VON prodrug facilitates slow prodrug dissolution and hydrolysis in biological matrices, a key mechanism for the extended duration of drug action [18,28]. In vitro cytotoxicity assay in macrophages confirmed NM2VON safety in phagocytic cells. Macrophages were used for in vitro safety assessment because they modulate drug absorption from the injection-site depot. Macrophages facilitate drug uptake and storage through their large cytoplasmic-to-nuclear ratio and phagocytic and proinflammatory functions. Monthly IM injections of NM2VON were well tolerated by mice and rats, enhancing drug delivery to the liver relative to the plasma. Targeting FXR agonists into the liver through lipophilic prodrug approaches while simultaneously restricting plasma drug exposure could maximize efficacy and lower side effects [32]. Given the limitations of our short-term PK data at a single dose, future studies will focus on detailed PK profiling and safety evaluations of NM2VON formulations administered at various dose levels and routes of administration. Safety evaluation will include serum lipid profiling since FXR agonists modulate lipid homeostasis. Upon absorption and dissolution from the particle, the M2VON ester prodrug is expected to hydrolyze rapidly into the pharmacologically active VON. Extensive hydrolysis studies on similar monomeric prodrug nanocrystals from our laboratory have demonstrated equal involvement of both enzymatic and chemical hydrolysis [18,33]. Future absorption, distribution, metabolism, and excretion (ADME) studies will be evaluated using the most thermodynamically stable polymorph of the M2VON prodrug. 

Progressive liver fibrosis is a common complication of HBV infection and a major contributor to liver-related diseases. HSC activation is one of the primary drivers of fibrogenesis [34]. In human LX2 cells, an immortalized human hepatic stellate cell line, NM2VON regulated liver fibrosis by significantly suppressing αSMA, TGFβ, and Col1A1 gene expression compared with VON treatment. We further validated this by identifying downstream signaling pathways, which showed that NM2VON downregulated TGFβ-induced SMO activation, the most downstream factor in Hh pathway activation. Our observations are consistent with prior studies showing that aberrant Hh pathway activation promotes HSC activation and collagen deposition during hepatic fibrogenesis [35]. The improved in vitro anti-fibrotic activity of NM2VON compared with VON likely reflects enhanced intracellular drug delivery and retention, since prodrugs are not pharmacologically active.

Monthly NM2VON treatment demonstrated anti-fibrotic efficacy in both HBV-negative and HBV-positive transgenic mice. CCl4-induced pro-fibrotic changes are a classic model of liver damage in experimental animals that recapitulate key features of liver fibrosis, including hepatotoxic injury, inflammatory cell infiltration, HSC activation, excessive collagen deposition, and upregulation of central profibrotic signaling pathways. These pathways include TGFβ and TIMP1 expression. Notably, NM2VON downregulated CCl_4_-activated fibrotic genes (mRNAs) responsible for collagen and ECM accumulation. Excessive ECM remodeling is associated with fibrosis [36]. NM2VON treatment decreased desmin, a fibrotic marker, levels in the liver tissue. AST levels were also decreased in NM2VON-treated mice, underscoring the potential of the LA formulation to protect the liver from CCl_4_-induced toxicity. In HBV transgenic mice, the antifibrotic effects were not related to VON’s antiviral properties, as HBV DNA levels in the CCl4-only and CCl4 + NM2VON groups were comparable, suggesting distinct antifibrotic mechanisms. It is also worth noting that the HBV-replicating mice used in this study do not develop an antiviral response. Together, these findings suggest that combinations of potent anti-HBV therapies with NM2VON could potentially improve viral suppression and regression of liver fibrosis in patients with chronic HBV infections. Although the mouse experiments were not designed to compare the efficacy of VON and NM2VON, our in vitro studies compared their ability to block/reduce profibrotic activation following TGFβ exposure. Although NM2VON-treated cells showed significantly greater αSMA attenuation, both treatments suppressed Col1A1 mRNA similarly. These findings suggest efficient conversion of M2VON prodrug into the pharmacologically active VON. 

Extrahepatic manifestations, including pulmonary disorders, are known complications of HBV infection [37,38,39]. In this study, we observed pulmonary fibrosis progression in mice exposed to CCl_4_. Fibrotic deposition in the lung is a consequence of activated fibroblasts and profibrotic cell differentiation, producing increased levels of collagen, an essential component of the ECM of fibrotic foci [40]. Notably, monthly NM2VON administration reduced CCl_4_-induced collagen deposition in the lung tissues of HBV-transgenic mice by approximately 50%, as assessed by Masson’s trichrome staining. An essential mechanism by which NM2VON reduces pro-fibrotic mRNA activation in liver tissue is by decreasing TGFβ levels through reduced PKC activation [41]. PKC activation is required to produce TGFβ, a major stimulator of fibroblast proliferation. Similarly, TGFβ can stimulate PKC activity, forming an autocrine activation loop. We observed reductions in TGFβ expression and PKC activation in the liver and lung tissues of NM2VON-treated mice. Our data suggest that NM2VON treatment reduces fibroblast activation and proliferation and decreases ECM production by inhibiting PKC and TGFβ secretion. Mice treated with NM2VON were protected from CCl_4_ pulmonary fibrosis, as characterized by limited alveolar wall thickening, fibroblast proliferation, and cell migration. Notably, NM2VON attenuated chronic CCl_4_-induced fibrosis in both the liver and lung. Thus, the spectrum of protective NM2VON anti-fibrotic effects is broad and not specific to a particular organ.

## 5. Conclusions

NM2VON, an LA injectable prodrug nanosuspension, was developed to improve VON’s apparent half-life and improve the treatment outcomes of progressive fibrosis. Notably, monthly IM injections of NM2VON into transgenic mouse models of tissue fibrosis and HBV infection attenuated both liver and lung damage. Our data demonstrates that NM2VON decreases HSC activation. These promising proof-of-concept preclinical findings support further evaluation of NM2VON as a potential LA treatment for liver and lung fibrosis.

## Data Availability

The original contributions presented in this study are included in the article/Supplementary Material. Further inquiries can be directed to the corresponding authors.

## Supplementary Materials

The following supporting information can be downloaded at: https://www.mdpi.com/article/10.3390/biom16101443/s1, Figure S1. Proton NMR of M2VON; Figure S2. Carbon NMR of M2VON; Figure S3. Body weights of NM2VON-treated and control mice; Figure S4. Plasma and Liver drug distribution in Sprague Dawley (SD) rats; Figure S5. Body weights of NM2VON-treated Sprague Dawley (SD) rats; Figure S6. Tissue Histological assessment of NM2VON-treated Sprague Dawley (SD) rats; Figure S7. Serum Chemistry Assessment; Figure S8. Original WB images. Table S1. Complete blood count (CBC) Analysis.
