# Supplementary material for: A Long-Acting Farnesoid X Receptor (FXR) Agonist Prevents Fibrosis
Source: Biomolecules. 2026 Oct 3;16(10):1443. doi: 10.3390/biom16101443 (PMC13702190; doi:10.3390/biom16101443)
Supplement: Supplementary file 1 [file biomolecules-16-01443-s001.zip › biomolecules-4557243-supplementary.pdf]

Supplementary Materials for

A Long- Acting Farnesoid X Receptor (FXR) Agonist Prevents Fibrosis Progression in a Model of Chronic Liver Injury

Ashrafi Sultana et al.

* Corresponding Authors: Benson J. Edagwa, e-mail: benson.edagwa@unmc.edu; Larisa Poluektova: lpoluekt@unmc.edu and Natalia Osna, e-mail: nosna@unmc.edu.

This file includes:

Figures. S1 to S8
Tables S1

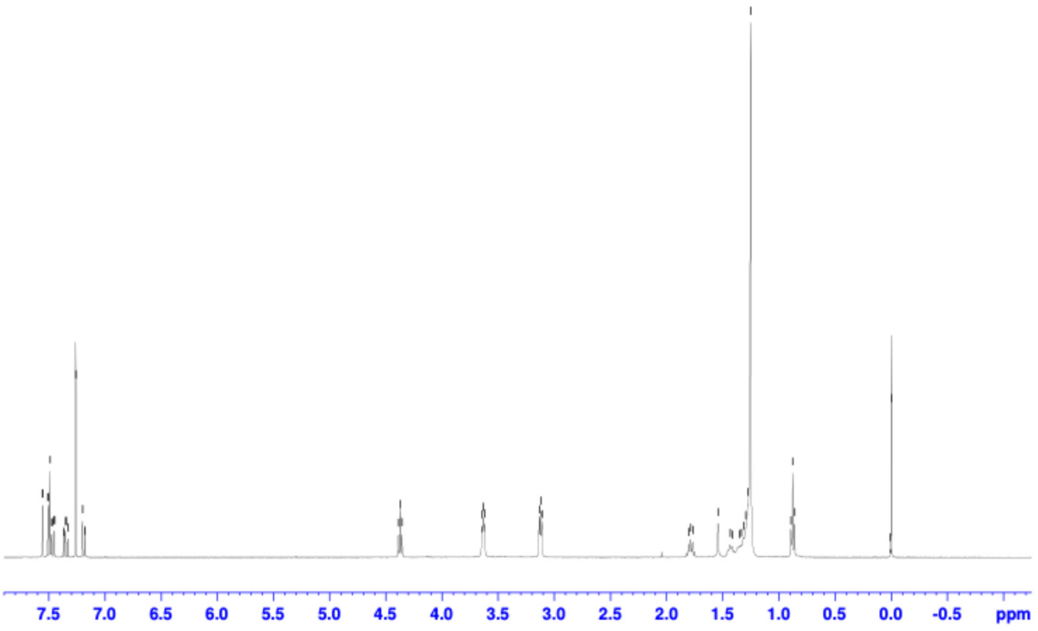

Figure S1. ^{1}H NMR Spectrum of M2VON in $CDCl_3$. Structural purity of M2VON was determined by ^{1}H NMR on a Bruker Avance III HD spectrometer (500 MHz, 11.7 T; Billerica, MA, USA). The experiments were repeated at least 5 times with equivalent results.

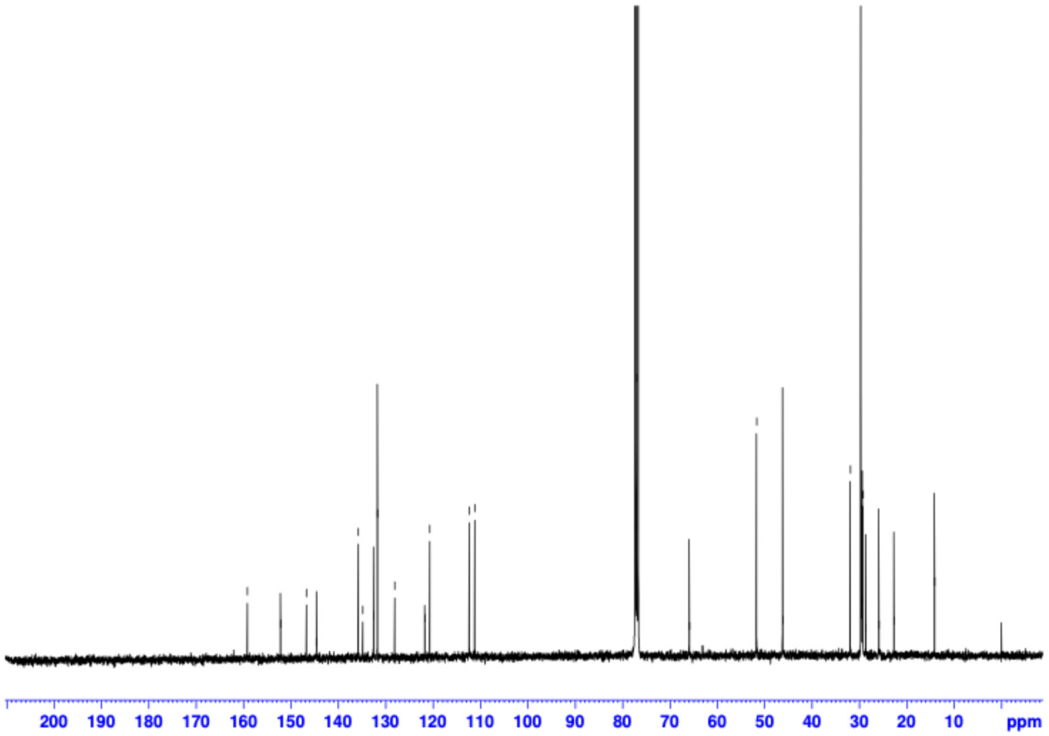

Figure S2. ^{13}C NMR Spectrum of M2VON in $CDCl_3$. The spectrum was recorded on a Bruker Avance III HD spectrometer (125 MHz, 11.7 T; Billerica, MA, USA). The experiments were repeated at least 5 times with equivalent results.

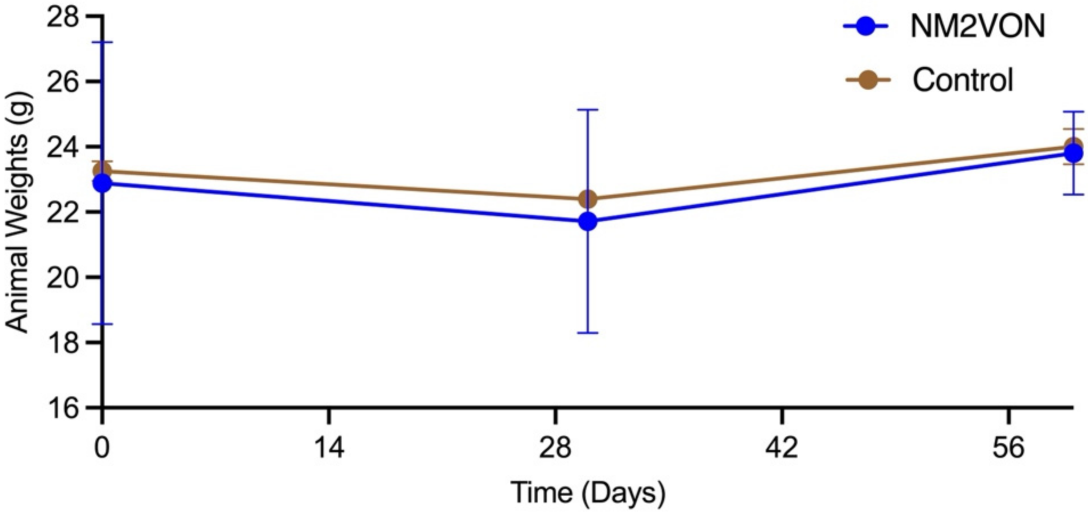

Figure S3. Body weights of NM2VON-treated and control mice. C57BL/6 male mice received the first NM2VON intramuscular injection (IM) at 200 mg/kg NM2VON, and five days later received twice-weekly intraperitoneal injections of 1 μg/g body weight of CCl4 in corn oil. The animals received subsequent NM2VON IM doses of 200 mg/kg in weeks 5 and 10, and an equivalent volume of the test article, as is standard for ISR studies. Drug levels were determined by LC-MS/MS, and data are presented as mean ± SEM. CCl_4 injections were continued until week 12, followed by animal sacrifice

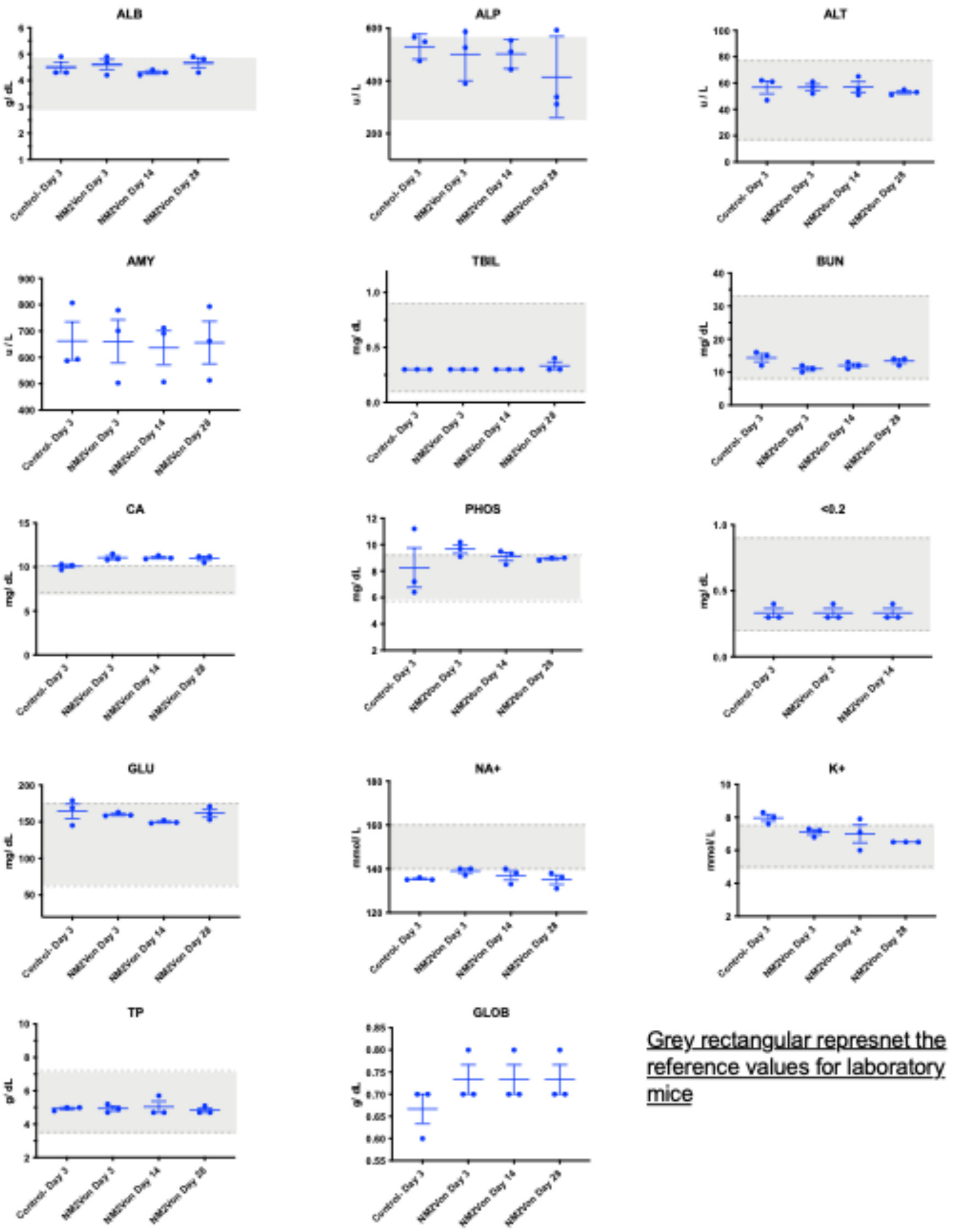

Figure S4. Serum Chemistry analysis of NM2VON-treated Sprague-Dawley Rats. Serum chemistry was recorded following a single intramuscular injection of 189-200 mg/kg NM2VON. Grey regions represent reference ranges obtained from the UCLA Division of Laboratory Animal Medicine.

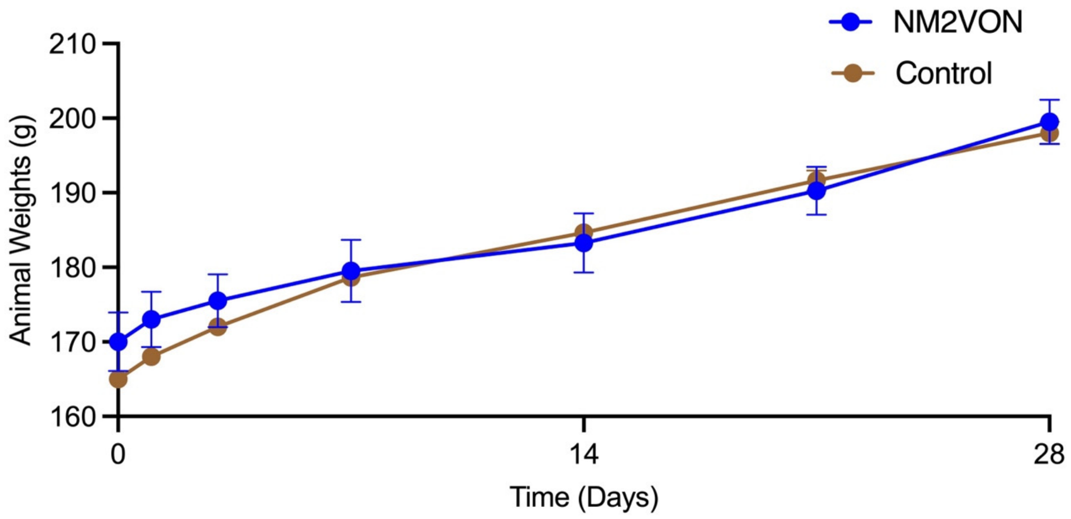

Figure S5. Body weights of NM2VON-treated Sprague-Dawley rats. The animal's body weight was recorded following a single intramuscular injection of 189-200 mg/kg NM2VON.

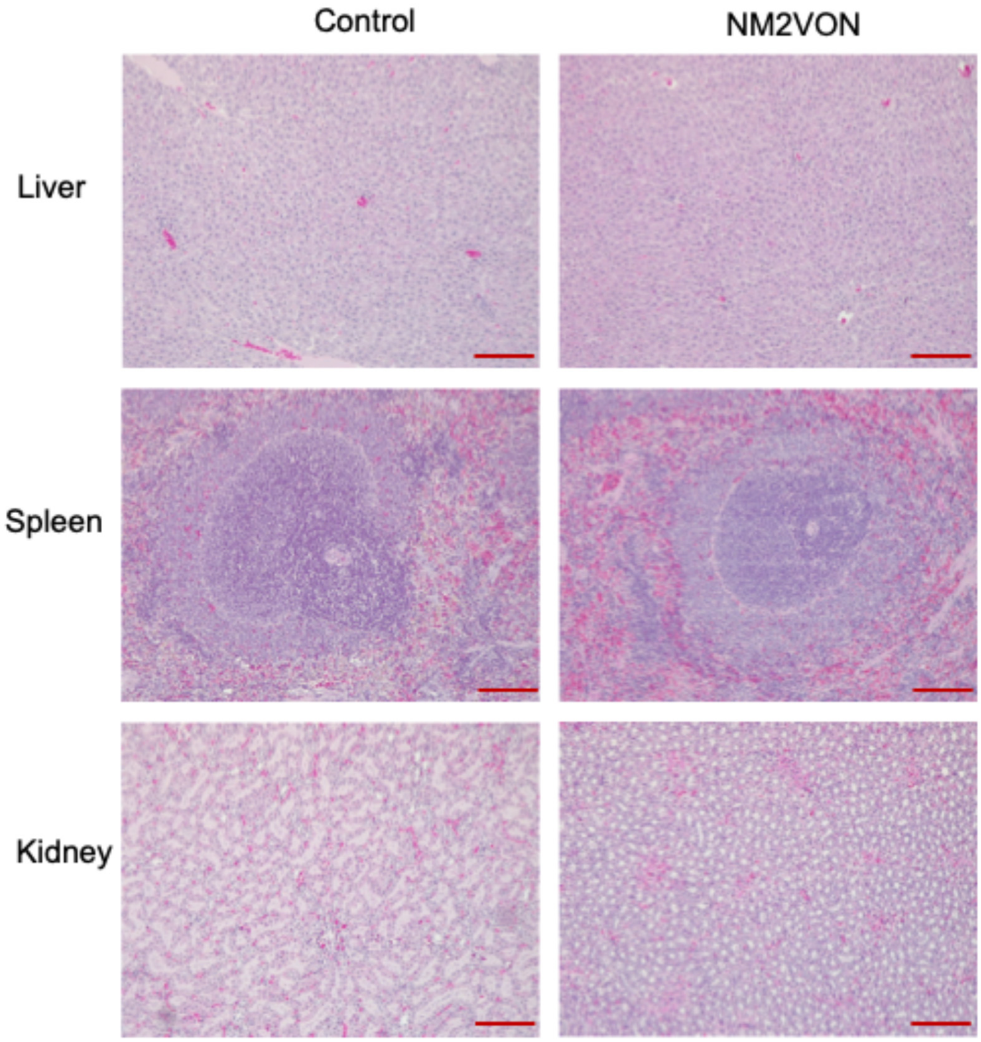

Figure S6. Tissue Histological assessment of NM2VON-treated Sprague-Dawley rats on day 28. H&E staining of liver, spleen, and kidney tissues on day 28 following intramuscular administration of NM2VON. The tissue sections for treated and control animals showed normal histology.

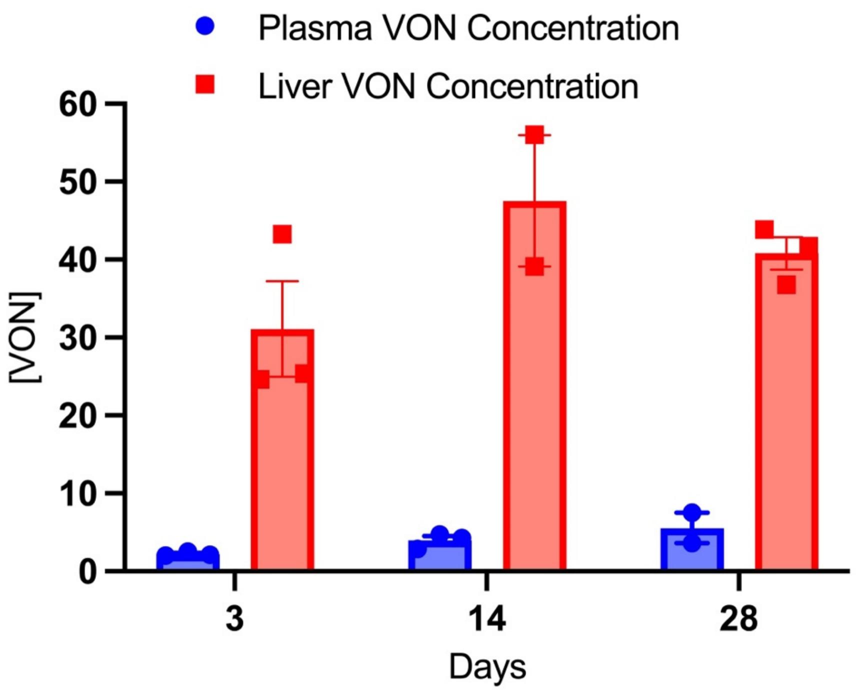

Figure S7: **Plasma and Liver drug distribution in SD rats**. Plasma (blue) and Liver (red) VON concentration in injection site evaluation study SD rats (N=3 per time point) after a single intramuscular injection of NM2VON at a dose of 189-200 mg/kg. All the animals (weighing between 250 and 300 g) received an equivalent volume of the test article, as is standard for ISR studies. Drug levels were determined by LC-MS/MS, and data are presented as mean ± SEM

	Day 3		Day 14		Day 28	
	**Control**	**treatment**	**Control**	**treatment**	**Control**	**treatment**
WBC (10^9 cells/L)	10.83	10.78	10.71	8.30	9.10	8.50
LYM (10^9 cells/L)	7.90	7.10	7.15	5.21	6.30	5.54
MON (10^9 cells/L)	0.23	0.21	0.25	0.27	0.20	0.22
NEU (10^9 cells/L)	2.49	1.97	1.98	1.76	1.90	1.69
LYM% (% of WBC)	85.3	76.5	80.8	73.4	72.8	74.6
MON% (% of WBC)	1.3	1.6	1.7	2.0	1.4	1.6
NEU% (% of WBC)	27.1	25.0	24.4	18.3	25.3	22.2
RBC (10^12 cells/L)	9.30	7.91	8.67	8.12	8.19	7.88
HGB (g/dL)	17.3	17.6	18.3	18.2	15.7	16.7
HCT (%)	53.30	55.10	52.43	43.90	49.10	48.73
MCV (fL)	61	61	63	65	58	60
MCH (pg)	18.4	19.0	18.5	17.3	17.4	17.1
MCHC (g/dL)	34.4	33.9	32.7	26.5	29.8	29.2
RDWc (%)	23.3	21.1	21.4	18.3	19.0	18.8
PLT (10^9 cells/L)	656	505	576	527	671	579
PCT (%)	8.50	9.08	9.09	8.80	8.10	8.67
MPV (fL)	0.5	0.7	0.6	0.4	0.5	0.5
PDWc (%)	36.4	33.8	34.7	33.1	31.9	33.9

Table S1. Complete blood count analysis of NM2VON-treated Sprague-Dawley rats. A complete blood count was recorded following a single intramuscular injection of 189-200 mg/kg NM2VON.

Uncropped Western blots used in the paper:

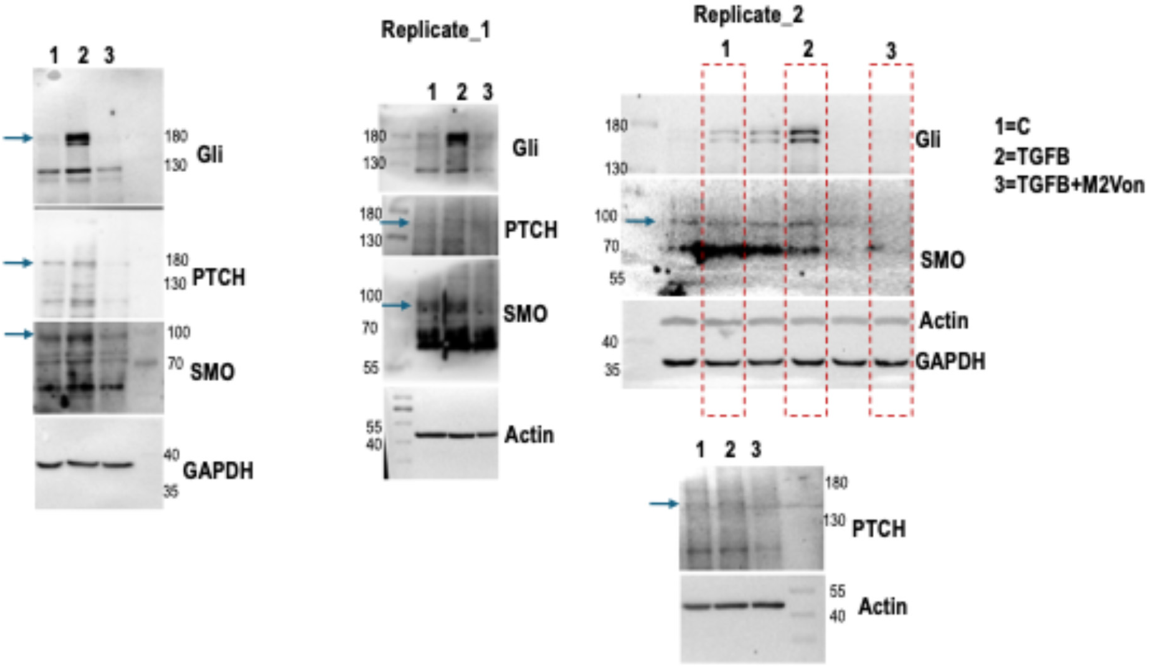

Figure S8: Western blots from replicate experiments showing the effect of NM2VON on Hh pathway proteins. LX2 cells were seeded at 400,000 cells/well and treated with NM2VON (100 µM) for 8 h before the addition of TGFβ (10 ng/mL). After 48 h, cells were harvested for protein analysis by Western blotting. GAPDH or actin served as an internal control.
